# Nanosecond photochemically promoted click chemistry for enhanced neuropeptide visualization and rapid protein labeling

**DOI:** 10.1038/s41467-019-12548-0

**Published:** 2019-10-16

**Authors:** Gongyu Li, Fengfei Ma, Qinjingwen Cao, Zhen Zheng, Kellen DeLaney, Rui Liu, Lingjun Li

**Affiliations:** 1 School of Pharmacy, University of Wisconsin-Madison, Madison, WI 53705 USA; 2 Department of Chemistry, University of Wisconsin-Madison, Madison, WI 53705 USA

**Keywords:** Mass spectrometry, Mass spectrometry, Chemical tools

## Abstract

Comprehensive protein identification and concomitant structural probing of proteins are of great biological significance. However, this is challenging to accomplish simultaneously in one confined space. Here, we develop a nanosecond photochemical reaction (nsPCR)-based click chemistry, capable of structural probing of proteins and enhancing their identifications through on-demand removal of surrounding matrices within nanoseconds. The nsPCR is initiated using a photoactive compound, 2-nitrobenzaldehyde (NBA), and is examined by matrix-assisted laser desorption/ionization-mass spectrometry (MALDI-MS). Benefiting from the on-demand matrix-removal effect, this nsPCR strategy enables enhanced neuropeptide identification and visualization from complex tissue samples such as mouse brain tissue. The design shows great promise for structural probing of proteins up to 155 kDa due to the exclusive accessibility of nsPCR to primary amine groups, as demonstrated by its general applicability using a series of proteins with various lysine residues from multiple sample sources, with accumulated labeling efficiencies greater than 90%.

## Abstract (web summary)

Mass spectrometry-based quantitative proteomics aim to identify and quantify proteins from complex biological samples. Here, the authors developed a method for simultaneous high-throughput protein labelling and on-demand matrix removal within nanoseconds.

## Introduction

In systems biology and biomedical research, mass spectrometry (MS)-based quantitative proteomics has become a premier tool, whereby the increasing development of labeling techniques has substantially enhanced comprehensive identification and quantitation of proteins from complex biological samples^1,2^. Of note, although great advancements have been made along with the continuous progress in quantitative tag designs and widespread applications, the interrogation of those protein structures is largely, if not completely, conducted separately. Traditionally, proteomics MS^3–6^ and native MS^7^, especially in conjunction with ion mobility setups^8,9^, are considered as representative strategies for comprehensive protein identification and structural probing, respectively. However, they employ relatively independent workflows despite a few recent attempts to combine the synergies between them^7,10^. Consequently, comprehensive protein identification and concomitant structural probing of these proteins have not yet been accomplished simultaneously. However, the ability to combine and integrate these workflows is critically important to better understand structure-function relationship.

Therefore, it is highly desirable to develop a rapid, in situ and combinatorial regime with dual utilities for enhancing identification and enabling protein structural probing. Notably, ambient MS^11–13^ strategies enable direct analysis of biomolecules, e.g., primarily small molecules, from multiple sample sources with minimal sample preparations. Ambient MS utilizes various strategies^11,13–17^ to remove interfering matrices in situ surrounding the target biomolecules. Though only limited successful attempts have been seen during past decade of ambient MS for large proteins^14–18^, it is promising to bridge the gap between sample preparation-dependent protein identification and rapid, reliable structural probing with the concept of ambient MS. This would require effective design of rapid and efficient ambient MS probes that can access protein structures.

Generally, analytical separation, including microscale electrophoresis^15,18^, is an efficient strategy in many biological and chemical analyses of various target molecules, especially for peptides and proteins from complex biological samples^19,20^. To improve the separation efficiency and real sample applications, in situ analytical separation has been developed at microscale and even nanoscale in both time and space^15,17–19^. Photochemical microscale electrophoresis (PME) induced by a confined photochemical reaction has been achieved by laser irradiation of a photoactive compound, 2-nitrobenzaldehyde (NBA)^21^. Meanwhile, microscale thermophoresis (MST) has been extensively investigated where the separation is based on the directed movement of molecules along temperature gradient^22–26^. Both PME and MST have shown great potential in separation of biomolecules via fluorescence measurements.

The comprehensive identification and quantitation of proteins are more frequently and efficiently enhanced by tagging chemistry. As almost all peptides and proteins bear highly reactive primary amine groups, chemical tags that are primary amine-reactive have grown to be dominating labeling reagents, as demonstrated by the widespread and commercialized labeling reagents for MS-based proteomics including tandem mass tag-^27^ and isobaric tags for relative and absolute quantitation^28^. To further improve labeling efficiency, throughput and cost-efficiency, our lab has also developed a series of primary amine-reactive labeling reagents since 2009 with the first report on *N*,*N*-Dimethyl Leucine tags^3,29–31^. Further improvements in the tagging chemistry are still required for MS-based biochemical applications, including improvements in versatility, cost, labeling efficiency and throughput. In parallel, high-throughput tagging chemistry with commercialized reagents has been achieved through laser-induced in situ/in-source tagging^32^. In addition, offline tagging chemistry was also used for matrix derivatization to enhance protein detection during matrix-assisted laser desorption/ionization (MALDI)-MS^33^. The versatility of in-source tagging chemistry utilized in various sample sources has shown its unique advantages without or with minimal synthesis processes.

**Fig. 1:**
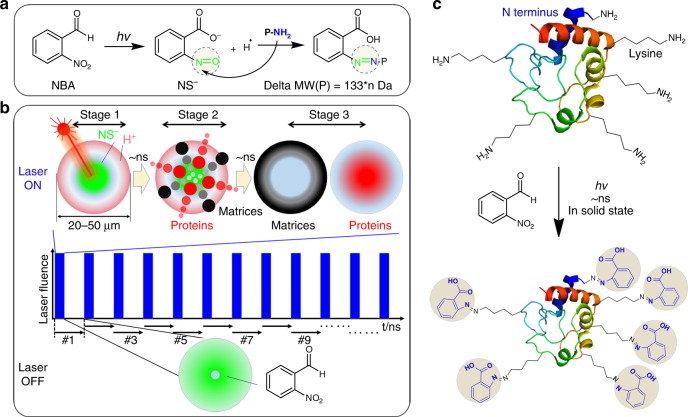
Nanosecond photochemical reaction (nsPCR). **a** Nanosecond photochemistry on 2-nitrobenzaldehyde (NBA) creating reactive 2-nitrosobenzoic anion (NS^−^) for the establishment of a localized micro-electric field and the labeling of primary amines in a protein. Tagging one NS^−^ results in a mass shift of 133 Da for proteins of interest. **b** On-demand three-stage matrix removal regulated by laser ON/OFF switch: Stage 1, laser irradiation establishing micro-electric field thermal gradient; Stage 2, molecular movement driven by localized PME and MST; Stage 3, the charged proteins stay near the center of laser spot while small matrices travel outside—they are thus separated within 3–5 ns and 20–50 μm. Furthermore, the electroneutral biomolecules tend to re-distribute along the thermal gradient upon laser irradiation. Both types of biomolecules can also be cleaned-up by a proton-matrix competition-related potential mechanism. **c** Schematic illustration of 2-nitrobenzaldehyde (NBA)-based nsPCR for large protein chemical labeling through nanosecond tagging surface accessible amine groups at the N termini and lysine residues

As noted, click chemistry has found a wide range of fascinating applications in chemistry, biology and materials including the biorthogonal ligation of proteins along with mechanistic explorations^34–37^. Recently, Gouin group reported an electrochemical method of tyrosine reactive click reaction for the conjugation of native proteins^38^. Gaunt group has also recently extended the protein bio-conjugation toolbox with a methionine-selective, quick tagging chemistry^39^. Inspired by the collective effects of in-source tagging, PME and MST designs, we herein develop a versatile nanosecond PhotoChemical Reaction (nsPCR)-based ambient MS probe (Fig. 1) by means of photochemically promoted click chemistry. This ambient MS probe can not only facilitate on-demand removal of surrounding matrices at nanosecond time scale and a few tens of micrometer scale, but also enable rapid, efficient and high-throughput tagging of these large proteins. Meanwhile, the photochemically promoted click chemistry and the matrix removal effects are directly observed with label-free MS in real time, whereby molecular identifications reveal the potential of nsPCR serving as an alternative ambient MS probe for a wide range of proteins, enabling comprehensive identification and in situ chemical labeling.

## Results

### Design of nsPCR

Our design relies on the nanosecond laser irradiation of photoactive NBA and its photochemical product (2-nitrosobenzoic anion, NS^−^). NS^−^ then reacts with free primary amine groups that are spatially accessible in proteins (Fig. 1a, b), and the differential distribution of NS^−^ and H^+^ establishes a fast-pulsed electric field to separate target proteins from small molecule-like matrices (Fig. 1c), in an on-demand manner with a confined lateral environment of about 20–50 μm. While several prior studies demonstrated the chemical nature of the photochemically promoted click chemistry, the principle of electrophoretic desalting/matrix removal has also been involved in multiple processes^15,17,40–42^.

High-beam millisecond synchrotron X-rays^43^ and nanosecond laser (3–5 ns, 266 nm, 30 Hz)^44,45^ have been previously used for residue oxidation-based solution protein surface mapping. Herein, we first adapted a nanosecond solid-state Nd:YAG laser with a relatively gentle nature (2 ns, 355 nm)^46,47^, to initiate photochemical reactions of NBA in a sub-atmospheric pressure (Sub)AP-MALDI source coupled to an Orbitrap instrument. A similar laser system in vacuum MALDI platform in a time-of-flight (ToF) instrument (Bruker RapifleX, more details in Online Methods) was then employed specifically for large protein structural probing. In our system, side reactions like extensive oxidation of amino acid residues were not frequently observed (see more details below in lysine labeling spectra), due to the well-controlled soft nature of rapidly pulsed (up to 10,000 Hz) laser generation and solid-state protein crystals. This feature can be beneficial for applications such as native-state or native-like structural elucidation of proteins/protein complexes without perturbations to original proteoforms induced by extensive external oxidation.

### Enhanced neuropeptide analysis via nsPCR

The on-demand removal of surrounding matrices would facilitate the structural probing of peptides/proteins from various sample sources. As a first step, the matrix removal enabled by nsPCR is linked to the creation of NBA-based micro-electric field and the simultaneous thermophoresis effect. To date, various desalting strategies have been extensively reported including electrophoresis^17,40^, micro-extraction^19,48^, recrystallization^14^, hydrophobicity regulation^49^, and even solution additive modification^50,51^. In this study, we designed an on-demand matrices-removal strategy based on the nsPCR of NBA. For charged large proteins, this matrices-removal strategy at nanosecond time scale and micrometer spatial scale relies on the three-stage nsPCR process. Stage 1: laser irradiation, which produces NS^−^ and H^+^, then establishes a localized microscale electric field; Stage 2: molecular movement driven by localized microelectrophoresis and thermophoresis, small matrices have a higher mobility rate than peptides/proteins; Stage 3: as a result, the peptides/proteins stay near the center of laser spot while small matrices travel outside, which leads to separation of peptides/proteins and matrices.

To ascertain the more generally applicable mechanism involved in matrix removal by NBA-based nsPCR, we first characterized the ion suppression effects and matrix adduction effects in AP-MALDI. Neuropeptide mixtures were used to observe the frequency- and laser energy-dependency of ion suppression effects and matrix adduction effects. As shown in Supplementary Fig. 1, upon increasing the laser energy, both ion suppression effects and matrix adduction increase. Surprisingly, upon increasing the laser frequency, ion suppression increases but matrix adduction decreases. In addition, in order to further verify the matrix removal effects, we also performed a series of experiments with NBA in PBS (1×) buffer solution at two pH conditions (pH 3 and pH 7). Results in Supplementary Fig. 2 suggest a pH-dependent matrix removal by NBA-based nsPCR, where the clean-up is more effective at lower pH conditions. Notably, the NBA-based nsPCR has also significantly simplified mass spectra compared with control group of tryptic mixtures (Supplementary Fig. 3), where few sodium/CHCA adducts were observed in several NS^−^-tagged peptides. Benefiting from the thermal and micro-electrophoretic separation initiated by the laser irradiation, sodium and CHCA adducts in Supplementary Fig. 3b were efficiently eliminated, especially for peptides **3** and **4**, in which the matrices (including Na^+^ and CHCA) bound ratio decreased from ~64 to ~25% and from ~45 to ~27%, respectively. Taken together, we therefore believe the matrix removal is not only linked to localized microelectrophoresis and thermophoresis which have been extensively observed by fluorescence measurements^22–26^, but is also related to the pH-jump induced by NBA dissociation. Charged biomolecules will be separated preferentially with the drive of localized electric field, while electroneutral biomolecules tend to re-distribute along the thermal gradient upon laser irradiation. Both types of biomolecules can also be cleaned-up by a potential mechanism related to proton-matrix competition, as the matrix adducts are effectively decomposed along with the increase of proton level (e.g., low pH conditions). Under this mechanism, peptide/protein protonation is preferentially directed during desorption and ionization, and matrix effects are therefore alleviated or removed.

The on-demand matrix removal feature of NBA-based nsPCR, interestingly, finds an innovative application in enhancing the creation of a detailed map of brain neuropeptides and lipids. As we know, the brain is the central nervous system of animals and direct visualization of the spatial distributions of neuropeptides and lipids helps better understand their regulatory roles for various activities. MS-based imaging (MSI), including atmospheric pressure (AP)-MALDI-MSI, has been an attractive alternative for sensitive visualization of those biomolecules directly from brain tissue section with high resolution^47,52–54^. Although the recently reported AP-MALDI-MSI showed improvements in lateral resolution^53^, it often suffers from limited sensitivity due to distinct ion suppression effects^54^. Among all the contributing factors for ion suppression effects, the most significant factor contributing to poor signals seen in AP-MALDI-MSI was due to co-existing small molecule-like matrices (including the matrix for MALDI and other organic salts).

**Fig. 2:**
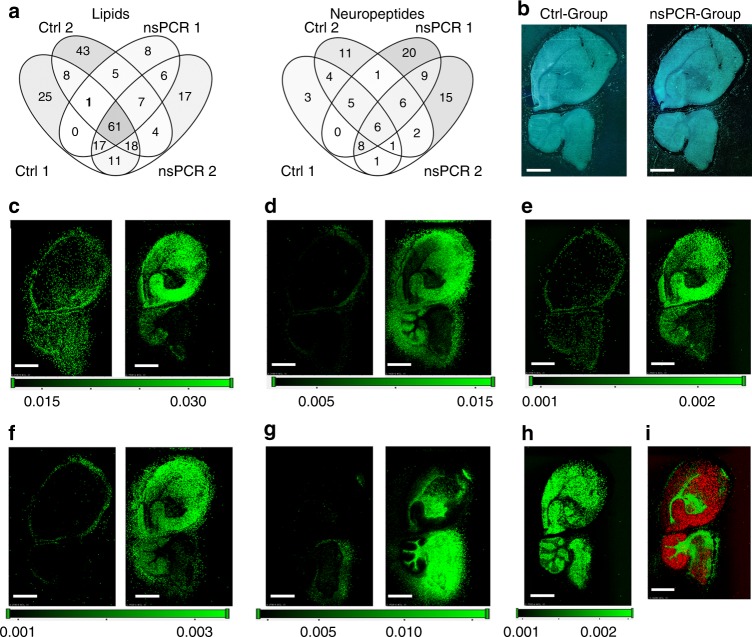
Enhanced neuropeptide visualization via nsPCR. **a** Venn diagrams showing two sets of comparative data for both lipid and neuropeptide identifications with and without NBA treatments. **b** Optical images of mouse brain for control and experimental group with NBA. **c** Direct comparison of putative neuropeptide ADKNFLRFamide ([M + H]^+^, *m/z* 1009.568) without (left panel) and with NBA treatment (right panel); **d** APQRNFLRFamide ([M + H]^+^, *m/z* 1147.650); **e** RKPPFNGSIFamide ([M + K]^+^, *m/z* 1199.610); **f** RSAEGLGRMGRL ([M + K]^+^, *m/z* 1340.660). **g** Direct comparison of lipid (triacylglycerol, [TAG(52:9) + Na]^+^, *m/z* 867.651) distributions in mouse brain between control and labeled group with NBA. **h** Typical distribution of lipid (monomethyl-phosphatidylethanolamine, [MMPE(44:10) + 34 + Li]^+^, *m/z* 878.645) with NBA. **i** Overlay of lipid (phosphatidylcholine, [PC(36:1) + H]^+^, *m/z* 788.615, red) and neuropeptide ([SKNYLRFamide + H]^+^, *m/z* 926.522, green) ion images. All images were obtained with *m/z* tolerance of 5 ppm. Scale bar, 2 mm. Step size, 50 μm

For example, neuropeptide identifications (IDs) are often affected by those small molecule-like matrices, which would significantly suppress the ionization and desorption of neuropeptides. In this study, we tested our strategy on two consecutive mouse brain tissue sections, one with NBA after matrix application and one without NBA as the control group. Combined data from two sets of experimental and control groups (Fig. 2a) revealed the similar lipid identifications as indicated by the highly shared lipid IDs (>62%, 124/200 for normal MSI or 124/155 for NBA-equipped MSI). On the other hand, improved identifications of neuropeptides were achieved (Fig. 2a) on the NBA-modified tissue section, as revealed by the much higher number of total neuropeptide IDs (74) compared with the control group (48). Notably, from the combined datasets of two groups, unique IDs from NBA-modified tissue (44) was much higher than that of the control group (18). According to our hypothesis, NBA-based nsPCR can remove matrices through on-demand established microelectrophoresis and thermophoresis, enabling the improved IDs of neuropeptides without sacrificing the lipid identifications as lipids bear much higher abundances and ionization efficiency.

We then examined the effects of NBA on distribution patterns of lipids and neuropeptides directly from two consecutive brain tissue sections. Optical images in Fig. 2b indicated that structural morphology of brain sections after NBA application was well maintained. Selective ion images were shown in Fig. 2c–i and overall, much higher quality images were obtained with the assistance of NBA-based nsPCR. Imaging contrast was significantly improved for almost all ion maps as shown. Some of the neuropeptides were detected under both conditions. However, the control groups exhibited more random and diffused lateral distribution patterns (e.g., Fig. 2c, e) while NBA applied tissue section displayed more localized distribution patterns with better alignment with the anatomical structure. Differential analyte suppression by matrices has led to the false distribution pattern in such a heterogeneous tissue. As a result, ion images of the control group sometimes were not reflective of biological spatial distributions. To this end, the matrix removal enabled by NBA-based nsPCR through on-demand, nanosecond microelectrophoresis and thermophoresis accounted for the enhanced visualization of neuropeptides in mouse brain tissue. This feature would facilitate the reconstruction of mouse brain high-resolution structures based on the ion images as shown in Fig. 2i. To conclude from Fig. 2, our imaging data from the AP-MALDI-MSI platform show enhanced identification of neuropeptides in both IDs and spatial distribution patterns using NBA-based nsPCR.

### Highly efficient lysine labeling via nsPCR

**Fig. 3:**
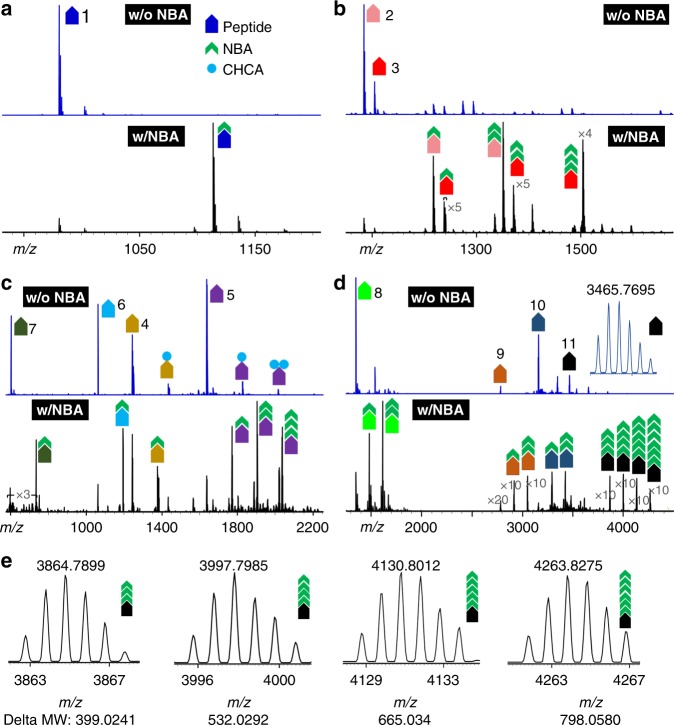
Highly efficient peptide labeling via nsPCR. A wide range of peptides (**a**–**d**, with increasing masses) with various K-residue numbers were tested using nsPCR labeling. Isotopic distributions of peptide 11 in **e** and insert in **d** confirmed the accuracy of the labeling results. w/o, without, w/, with. Individual peptide sequence information are listed in Table 1. Source data is provided in a Source Data file

We then evaluated the throughput and efficiency of this strategy by labeling solid peptide crystals pre-deposited on a MALDI plate (Fig. 3). With a series of peptide standards ranging from 599 Da to over 4000 Da, the comparative spectra as obtained from NBA-modified MALDI-MS measurements (Fig. 3) distinctly demonstrated the photochemically promoted click chemistry with a mass increase of 133 Da for one labeling tag. Multiple adducts with mass increases of (*n* + 1)*133 Da can be observed when there are various K-residue numbers (*n*) present in target peptides/proteins. Isotopic distributions of peptide **11** (Beta-Endorphin, human)-related peaks were presented in Fig. 3d, e, which confirmed three to six NBA molecules were attached as indicated by the mass shift from 399.0241, 532.0292, 665.0334 to 798.0580, respectively, revealed by high-resolution and high-accuracy measurements. Notably, simultaneous labeling of multiple peptides as shown in Fig. 3 also suggests the feasibility for high-throughput peptide and protein labeling applications.

Notably, labeling results in Fig. 3 suggested the issue with non-stoichiometric NBA tagging that leads to incomplete labeling. For example, peptide #8, substance P (RPKPQQFFGLMamide), shows two labeling peaks after NBA labeling as the peptide has two available labeling sites. It would be more beneficial for future applications to real biological samples if the labeling can occur with complete stoichiometry. To this end we then performed further optimization of labeling conditions using Substance P peptide as a model compound. As invoked by the pH-jump hypothesis, two sets of labeling experiments were performed on the peptide Substance P (RPKPQQFFGLMamide) at two pH conditions (pH 3 and pH 7) with PBS (1×) buffer solution, both of which were screened with a range of NBA concentrations from 0 to 50 mg mL^−1^. Results in Supplementary Fig. 4 indicated that peptide labeling by NBA-based nsPCR progressed in a pH-dependent manner: lower pH condition facilitated the complete stoichiometric tagging of peptides (>99% for two NBA-tagged peak). In addition to the matrix removal effects seen in the spectrum, this experiment shows a great potential for future real sample applications through pH adjustment to achieve an ideal complete stoichiometry.

Having collected the labeled spectra, quantitative evaluation of the NBA labeling efficiency was further performed through calculation of the relative intensity after NBA tagging compared with that without NBA reaction. Table 1 showcases the labeling efficiency for each of 11 tested peptides with various K-residue numbers. The labeling number fits well with the total number of K-residue and the N termini primary amine group. Thus, the data clearly indicate that the specificity of NBA tagging under nsPCR exclusively targets for N termini and K-residue primary amine groups. The nsPCR labeling efficiency can be greater than 60–90% for most peptides, except for the one with a P residue at N termini (such as peptide #4 in Table 1). The relatively low labeling efficiency for those exceptions likely originated from large steric hindrance of the proline side chain.

**Table 1:** Highly efficient peptide/small protein labeling via nsPCR

Entry	Sequence	Mass	K#	Label#	Notes	Labeling efficiency (%)^a^
1	HHYAHGFLamide	980.1 Da	0	1	Neuropeptide	91.43%
2	HIASLYKPR	1084.3 Da	1	2	Neuropeptide	75.56%
3	KHKNYLRFamide	1104.4 Da	2	3	Neuropeptide	68.11%
4	PDVDHVFLRFamide	1243.4 Da	0	1	Neuropeptide	40.81%
5	AG*CKNFFWKTFTS*C	1637.9 Da	2	3	Somatostatin	57.61%
6	RPPGFSPFR	1059.6 Da	0	1	Bradykinin	78.57%
7	FMRFamide	599.3 Da	0	1	Neuropeptide	93.88%
8	RPKPQQFFGLMamide	1347.8 Da	1	2	Substance P	68.80%
9	MLGNKRPGLSGLTLALSLLVCLGALAEA	2780.3 Da	1	2	Neuropeptide	73.07%
10	GWTLNSAGYLLGPHAVGNHRSFSDKNGLTS	3157.5 Da	1	2	Human galanin	68.45%
11	YGGFMTSEKSQTPLVTLFKNAIIKNAYKKGE	3465.0 Da	5	6	β-Endorphin	63.27%
12	A chain: GIVEQ^‡^C^Π^CTSI^‡^CSLYQLENY^§^CN	5807.5 Da	1	3	Human insulin	67.75%
	B: FVNQHL^Π^CGSHLVEALYLV^§^CGERGFFYTPKT					

The labeling efficiency listed at the last column is the overall labeling value, and the calculation functions are provided in Online Method section

*^,^^‡^Intra-chain disulfide bonds between cysteine residues. ^∏,^^§^Inter-chain disulfide bonds between cysteine residues

^a^The overall labeling efficiencies represent three replicates with RSD <5%

The next step is to demonstrate the direct reaction between primary amines from N termini and K-residue. To achieve this, we have synthesized two peptides, HIASLYKPR and AGHK_Boc_LL (the free amine at lysine was capped with tert-butyloxycarbonyl (Boc)-group). HIASLYKPR has two available sites (N termini and K-residue), while AGHK_Boc_LL only has one available site (N termini). Their representative tandem MS spectra were shown in Supplementary Figs. 5, 6. Both peptides are with good sequence coverage as indicated by the tandem MS spectra of apo-species before NBA labeling. Interestingly, for HIASLYKPR (Supplementary Fig. 5), the NBA labeling numbers for b_1_ to b_6_ ions were one, as all of them contained one N terminal free amine group. Notably the b_7_ ion was observed with two NBA molecules attached, which was in good accordance with its nature of having two free amine groups (one N terminus and one K-residue). Meanwhile, for all of the detected y ions derived from HIASLYKPR, only y_1_ and y_2_ ions were not tagged by NBA while the others were tagged with one NBA molecule. This result is also in line with the available amine numbers. As a second example, AGHK_Boc_LL contains a lysine residue but was capped with Boc protecting group. Consequently, although the peptide has one lysine residue, it contains only one single free amine group. As shown in the tandem MS spectrum (Supplementary Fig. 6), all of the b ions were tagged with one NBA molecule while all the detected y ions were free of NBA tagging. Taken together, the tandem MS results unambiguously demonstrated the primary amine-specific reaction by NBA, including N termini and lysine residues. The strategy of NBA-based nsPCR, however, is not only applicable for isolated peptide/protein standards, but also works for high-throughput and highly efficient amine labeling from complex mixtures. Cytochrome c (Cyt c) was first enzymatically digested with standard bottom-up proteomic procedures by using trypsin. Cyt c enzymatic digests (1 µg µL^−1^), all of which were first analyzed and identified by LC-MS/MS, were mixed with matrix CHCA (10 mg mL^−1^) in 1:9 (v/v) ratio. To initiate NBA-based nsPCR, the cyt c-CHCA mixture was further mixed with NBA (10 mg mL^−1^) in 1:1 (v/v) prior to co-deposition onto the MALDI plate. The control group of cyt c digests was mixed (the same 1:1 ratio) with solvents (ACN/EtOH/FA/H_2_O, 84/13/0.3/2.7). Representative mass spectra for Cyt c fragment labeling and control group without the use of NBA are shown in Supplementary Fig. 3b. Distinct labeling events can be tracked from the comparative spectra and, as calculated from the intensity ratio of labeled peaks to apo-peptide peaks, the labeling efficiency (Supplementary Fig. 3c) for various peptides can reach ~90%. The tagging specificity of N termini and K-residue primary amine group was also observed among all the identified Cyt c fragments as the number of NS^−^ tagging is strictly equal to the (*n* + 1), where *n* refers to the number of K residues. In addition to tryptic peptide mixtures, the NBA-based nsPCR has also shown its labeling capability for neuropeptide extracts from crab brains (Supplementary Fig. 7 & Supplementary Data 1).

### Rapid protein labeling via nsPCR

In addition to peptides, intact proteins can also be efficiently labeled using the photochemically promoted, nanosecond click chemistry-based tagging strategy. Human insulin, an important protein hormone that regulates blood glucose levels, with two chains linked by two inter-chain disulfide bonds and one intra-chain disulfide bond, was labeled with three NS^−^ tags and the overall labeling efficiency was >95% (Supplementary Fig. 8). Moreover, Cyt c, a model protein with highly conserved structure that plays multiple roles including the essential component of the electron transport chain in mitochondria, was selected as a typical example for larger proteins^55^. It was noted that Cyt c was almost tagged with nine NS^–^ groups (Supplementary Fig. 9), which appeared to occur in a protein structure-dependent manner as the nsPCR-labeled lysine residue number was well in accordance with that in unstructured sequence segments and N terminal amino group.

**Fig. 4:**
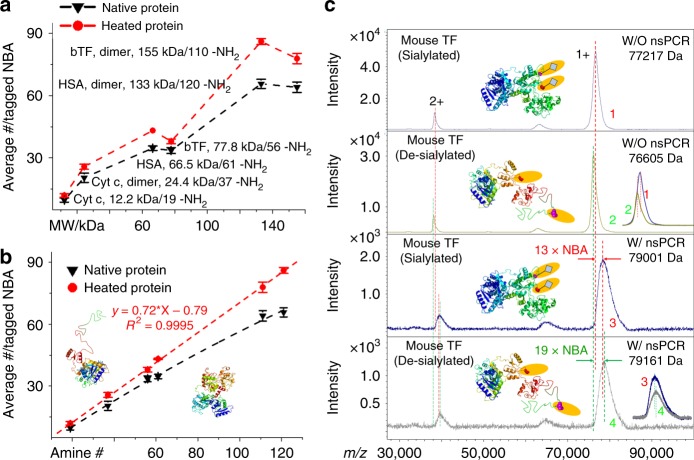
Large protein labeling via nsPCR. **a** Correlation between average number of nsPCR-crosslinked NBA and the molecular weight of large proteins. **b** Correlation between average number of nsPCR-crosslinked NBA and the total number of free amine groups on large proteins. Cyt c, cytochrome c; HSA, human serum albumin; bTF, bovine transferrin. The proteins in control group are preserved with 100 mM ammonium acetate, while unfolded proteins are obtained through heating in 100 mM ammonium acetate at 95 °C for 10 min. The error bars represent S.D. with *n* = 3 biologically independent experiments. **c** The structural effects of terminal sialylation on glycoprotein mouse transferrin (TF) probed by nsPCR. As a control (de-sialylated), two sialic acids (Neu5Gc, mass shift between peaks 1 and 2) on mouse transferrin are removed with sialidase under the native condition. The number of surface accessible amine group for sialylated (mass shift between 1 and 3) and de-sialylated (mass shift between peaks 2 and 4) transferrin is probed as 14.4 ± 1.1 (*n* = 3) and 20.7 ± 1.3 (*n* = 3), respectively. W/O, without; W/, with. Source data is provided in a Source Data file

NBA-based nsPCR can also serve as a rapid and efficient structural probe through probing the surface accessible free amine groups on large proteins (Fig. 4). To evaluate the versatility for structural probing via nsPCR, a series of large proteins with distinct spatial arrangements containing various domains and secondary structural elements were subjected to NBA-based nsPCR before and after heating treatment. Representative mass spectra of Cyt c, human serum albumin (HSA) and bovine transferrin (bTF) were shown in Supplementary Fig. 9, Supplementary Fig. 10, and Supplementary Fig. 11, respectively. On one hand, the raw mass spectra reveal distinct NBA-tagging chemistry on these large proteins with masses up to 155 kDa as indicated by the mass increments of both the natively preserved and the heating-unfolded proteins. On the other hand, the significantly higher mass shift of heating-unfolded proteins compared with natively preserved proteins indicated the increased exposure of free amine groups due to structural unfolding. Figure 4a showed the general trend of positive correlation between average numbers of labeled NBA molecules and the protein molecular weights, though bTF proteins slightly drop due to the reduced total numbers of amine groups.

We then plotted the average number of labeled NBA molecules, which is the same as labeled free amine groups, against the total number of free amine groups in proteins in Fig. 4b. The labeled amine group numbers on both natively preserved and heating-unfolded large proteins distinctly increase with the elevation of total amine numbers. It was observed with high-confident linear correlation between these two numbers for unfolded proteins with an R^2^ value of 0.9995. The fitting slope value of 0.72, rather than theoretical value of ~1.0, in Fig. 4b, indicates partial unfolding of these proteins due to the proteins being heated in native MS buffer of ammonium acetate (100 mM). The structural shielding effects on amine groups can be derived from the amine labeling ratios on natively preserved and heating-unfolded large proteins that are 50–60% (e.g., 34 out of 61 for natively preserved HSA) and 63–71% (e.g., 43 out of 61 for unfolded HSA), respectively. We exclude the possibility of low reactivity of NBA-based nsPCR because the labeling efficiencies on most peptides (Fig. 3 and Table 1) and small proteins (Supplementary Figs. 8 and 9) have been demonstrated to be higher than 90%. It should be noted, though it is not necessary to observe linear correlation on natively preserved proteins as differential structural shielding effects may exist amongst these proteins, a near-linear changing trend was also plotted for natively preserved proteins (Fig. 4b).

As a next step, to evaluate the labeling efficiency of NBA-based nsPCR, we first examined the surface accessible free amines in these proteins, Cyt c, HSA and bTF. A recently released software, NetSurfP 2.0, was employed as a functional analysis tool with a default exposure threshold of 25%^56^. The prediction results on three protein monomers are plotted in a bar graph (Supplementary Fig. 12) along with the NBA-based nsPCR labeling results. The comparative results on three protein monomers, interestingly, show that our nsPCR labeling numbers correlate ~50–60% of the predicted values (that are obtained with an exposure threshold of 25%). This observation suggests that nsPCR should unambiguously explore lysine residues on protein surface with an exposure degree of more than 40–50%. Furthermore, to ascertain the labeling efficiency of NBA-based nsPCR on large proteins, we also designed two sets of in-solution labeling experiments as control groups (Supplementary Figs. 13, 14). We found that NBA (Supplementary Fig. 14A) can tag up to 53 primary amine groups in solution, in comparison with 55 exposed (25%) lysine residues and ~35 NBA-tagged primary amine groups in the gas phase. This phenomenon indicates that in-solution labeling reaction is very close to complete stoichiometry and its labeling efficiency appeared to be higher than on-plate NBA-based nsPCR, although the latter technique should show great promise in the future upon further optimization (more details in Discussion part). The direct comparison between two different in-solution labeling reactions suggests the potential steric effect for relatively larger molecule NBA over formaldehyde in terms of approaching free amine groups in proteins especially for those buried inside protein domains. However, we cannot exclude the possibility of protein denaturing or unfolding that induces such higher labeling numbers in solution as of the organic solvents used and other structurally harmful reaction conditions.

Collectively, the strategy of NBA-based nsPCR has demonstrated its versatility in high-throughput and highly efficient labeling of peptides/proteins with masses ranging from 599 Da to over 155,000 Da, which may find expanding applications in protein structure-related research including serving as a rapid and high-throughput screening tool for inhibitor discovery. We herein then employ nsPCR to probe the structural effects of terminal sialylations on glycoprotein mouse transferrin (Fig. 4c).

Sialylation is present in a large portion (~80%) of glycoproteins which represents a unique terminal glycosylation form with negatively charged, acidic residues. While the sialylation of glycoproteins like transferrin explored in this study has been linked to multiple diseases including Alzheimer’s disease^57–61^ little molecular and structural basis is known for the effects of sialylation on glycoprotein 3D structures. Our nsPCR strategy, as shown in Fig. 4c, can rapidly distinguish and structurally probe the differentially sialylated forms of the mouse transferrin (59 theoretical amine groups from UniProt ID of Q921I1). De-sialylation of transferrin results in decreasing mass of the protein from 77.2 to 76.6 kDa but increased NBA tagging from 13 to 19 (Fig. 4c), indicative of an extended exposure of amine groups on protein surface upon removal of sialic acid. This observation may support the stabilizing effect of sialylation on glycoprotein transferrin structure, as negatively charged terminal sialic acids may change glycan orientations through extra steric hindrance or direct interaction with protein backbone^62^. Interestingly, only slight changes in structural signal (measured by collisional cross-section values) were observed from the more traditional gas-phase structural characterization tool, ion mobility-mass spectrometry (Supplementary Fig. 15). These intriguing results further highlight potential utility of the nsPCR strategy as a complementary tool for structural biology with unique capability to probe dynamic changes caused by protein post-translational modifications and improved sensitivity on protein structural changes.

## Discussion

Current MS-based proteomics and protein structural research efforts are frequently hindered by limited sample amount and protein structural loss or artifacts induced by multistep sample preparations. A delicate balance is often needed to enable confident identification and in situ protein structural characterization. While ambient MS strategies^11–13^ have been extensively utilized to study small molecules and less frequently involved in proteins^14–18^, only limited cases have been reported that allow in situ protein structural probing via ambient MS^63^. Our current study aims to bridge the gap between comprehensive identification and in situ structural interrogation of large proteins. We propose a new design of ambient MS probe with nsPCR-based click chemistry, with the potential capability to simultaneously enhance protein identification and to enable in situ interrogation of their structures.

Data in this study support the NBA-based nsPCR with dual utilities for both highly efficient, high-throughput protein structural probing via chemical labeling and on-demand matrix removal at nanosecond time scale within a confined lateral space of about 20–50 μm. The structural probing on large proteins is based on direct reactions between surface accessible primary amine groups and photochemically initiated NS^-^ radicals, which has been evaluated using multiple types of peptides and proteins from various sample sources, including the isolated peptides, small proteins and large proteins with masses ranging from 500 Da to 155 kDa, tryptic protein fragment mixtures and raw tissue extracts of crab brains. Therefore, we envision significant promise of this photochemically promoted, nanosecond click chemistry as a rapid and high-throughput structural probe for larger proteins even directly from living cells^15,18^. This new method can offer a supplementary or alternative method of choice for ion mobility-dominated tools in structural mass spectrometry realm^9,64–67^ and many other protein structure-related research pipelines, as the tagging chemistry has high specificity to surface accessible amine groups. Although we only demonstrated examples of structural probing by nsPCR strategy on isolated large proteins, this strategy should be readily extended to many other types of samples, as the laser-based method has been demonstrated to be a versatile, in situ sampling tool from liquid water^68^, cells^69,70^, and tissues^71^.

Compared with in-solution labeling, the NBA-based nsPCR strategy will certainly require more careful optimization to achieve comparable labeling results and in some cases, still suffering from non-stoichiometric labeling. In this incomplete tagging situation, especially for real biological sample applications with increased complexity, several problems are noted, including: (i) the signal intensity of a peptide can be further diluted, and (ii) the chance of overlap with other peptides increases, which can potentially interfere with the accurate protein and peptide identification. Therefore, more work is needed to further improve the performance and utility of the NBA-based nsPCR labeling approach for widespread applications. For example, in situ digestion could be employed to confirm the labeling sites of proteins. With the advantage and continuous advancements of high-accuracy/high-resolution mass spectrometers, this technique has the potential of localizing and quantifying the protein distribution patterns on tissues (if NBA tags with different isotopes can be synthesized). It should be noted that, NBA-based nsPCR on-plate labeling offers several complementary benefits: (1) significantly reduced labeling time, which can potentially be very useful for large-scale screening applications; (2) minimal sample preparation requirement, which benefits from both the matrix tolerance of MALDI ionization process and further improvement by NBA, and can be powerful for direct/in situ biological sample analysis; (3) versatility for imaging analysis, which is much more flexible and popular for MALDI-based on-plate technique; thus, the NBA-based nsPCR has excellent potential for on-tissue analysis while maintaining the visualization function; (4) economical single-step chemistry under ambient conditions, which means it only requires the use of low-cost NBA reagents and can happen at ambient condition (room temperature and atmospheric pressure) compared with in-solution labeling conditions that are often water-resistant, temperature- and pressure-sensitive, and require expensive reagents and multistep chemistry.

Notably, the current strategy possesses the inherent benefits of ambient MS method, that is, achieving rapid and in situ peptide/protein identification through removal of surrounding matrices. MSI results demonstrated improved creation of spatial maps of endogenous biomolecules (e.g., neuropeptides and lipids) from heterogeneous mouse brain tissue sections. Benefiting from the unbiased regional suppression through the alleviated matrix effects that might hinder the relative and absolute quantitation^72^, we anticipate improved in situ quantitative imaging analysis and on-tissue proteomics enabled by this nanosecond photochemistry reaction, which will further expand the utilities and applications of this laser-based chemical tagging approach.

### Online content

Any methods, additional references, Nature Research reporting summaries, source data, and statements of data availability are available online.

## Methods

### Chemical reagents

Methanol (MeOH), acetonitrile (ACN), acetic acid (AA), 2-nitrobenzaldehyde (NBA), and formic acid (FA) were purchased from Fisher Scientific (Pittsburgh, PA). The matrices of sinapic acid (SA) and α-cyano-4-hydroxycinnamic acid (CHCA) were purchased from Sigma-Aldrich (St. Louis, MO). Microscope glass slides were purchased from VWR international, LLC (Radnor, PA). Conductive indium tin oxide (ITO) coated glass slides were purchased from Bruker (Billerica, MA). Human insulin was purchased from Promega (Madison, WI, USA). Other protein samples (including cytochrome c from bovine heart, bovine serum albumin, human serum albumin and bovine transferrin) were purchased from Sigma-Aldrich (St. Louis, MO). Peptide standards (bradykinin, somatostatin II, angiotensin II, FMRF, FMRFamide-like peptide II (lobster)) were purchased from American Peptide Company (Sunnyvale, CA, USA). Amyloid beta peptides were purchased from Sangon Biotech Co., Ltd. (Shanghai, China). No further purifications were performed for all reagents. All solvents used in this study were of HPLC grade. Purified water (conductivity of 18.2 MΩ.cm) was obtained from Milli-Q Reference System (Millipore Corp., Bedford, MA, USA).

### Matrix sample preparation

SA and CHCA matrix were used in this study. Specifically, during standard spot analysis, 10 mg mL^−1^ CHCA (ACN:H_2_O:FA (v/v/v) 49.95:49.95:0.1) was used for peptides and small proteins, and 20 mg mL^−1^ SA (ACN:H_2_O:FA (v/v/v) 49.95:49.95:0.1) was employed for larger proteins. For MSI, the concentration of CHCA was 5 mg mL^−1^.

### NBA sample preparation

NBA was first dissolved in the mixed solvent of ACN/EtOH/FA/H_2_O (84/13/0.3/2.7, v/v/v/v). Unless stated otherwise, for spot analysis, a concentration of 10 mg mL^−1^ was used, while 5 mg mL^−1^ was used for imaging analysis. For each group of imaging analyses, CHCA was deposited under normal TM sprayer conditions as specified below, then NBA was sequentially deposited with gentle condition of 30 °C and gas pressure of 10 psi. The TM sprayer was tuned to deposit almost 1:1 for CHCA:NBA to the tissue.

### Crustacean neuropeptide extraction and identification

Animal experiments were performed following the institutional guidelines (University of Wisconsin‐Madison IACUC). Before sample collection, rock crabs *Cancer irroratus* were anesthetized in ice for 20 min before sample collection. Crab brains were dissected and immediately transferred to acidified methanol (MeOH/H_2_O/HOAc, 90/9/1, v/v/v), followed by storage in dry ice. Samples were stored in −80 °C freezer until further handling. Tissues were probe-sonicated inside a 4 °C cold room with a sonic dismembrator (Fisher Scientific, Pittsburgh, PA, USA) with a pulse of 8 s on and 15 s off for three times. After centrifugation at 4 °C for 15 min with 18,000 × *g*, the extraction supernatant was then transferred onto Microcon YM-30 30 K MWCO filters (pre-rinsed with ACN/MeOH/H_2_O, 20/30/50, v/v/v) and spinned for 20 min with 14,000 × *g* at 4 °C. The flow-through was dried down using a Savant SC 110 Speedvac (Thermo Electron Corporation, Waltham, MA, USA). After desalting with Agilent Omix C18 tips, the sample was dried down and reconstituted in 12 µL water (containing 0.1% FA) for LC-MS/MS analysis. The crab brain neuropeptide mapping was performed with a Q Exactive HF hybrid quadrupole-Orbitrap mass spectrometer (Thermo Fisher Scientific) coupled with a Dionex Ultimate 3000 UPLC system. The mobile phase A was 0.1% FA water with mobile phase B being 0.1% FA ACN. Each 2 µL sample was separated by a 75 µm × 15 cm in-house packed LC column with 1.7 µm, 150 Å, BEH C18 material from a Waters UPLC column (Waters, Milford, MA). LC conditions for peptide separation included: flow rate of 0.3 µl/min, gradient ramping from 3% solvent B to 30% solvent B over 40 min; the gradient was quickly ramped from 30% B to 75% B within 0.5 min and remained at 75% for 8.5 min; the gradient was then quickly ramped from 75 to 97% B within 0.5 min and remained at 97% for 8.5 min. For MS analysis, the mass resolution of full MS scan was set to be 60 K. The top 15 most abundant ions were selected for data-dependent fragmentation with normalized collision energy of 30% at a mass resolution of 15 K.

### Bottom-up LC-MS/MS analysis

Protein sample (cytochrome c) was digested with 1 µL of 0.5 mg mL^−1^ trypsin (Promega, WI, USA) at 37 °C for 17 h, and then desalted with Sep-Pak C18 cartridge (Waters) and dried down in vacuum. All experiments were performed using a Q Exactive-Orbitrap mass spectrometer (Thermo Fisher Scientific) coupled with Waters nanoAcquity UPLC system with 0.1% formic acid (FA) water as mobile phase A and 0.1% FA ACN as mobile phase B. Samples were reconstituted in 0.1% FA, 3% ACN and loaded onto the fabricated column (150 mm × 75 µm, 1.7 µm). The gradient started with 100% A and was linearly ramped to 30% B in 120 min. The gradient was then ramped to 75% B in another 0.5 min and kept for 10 min, later ramped to 95% B in another 0.5 min and kept for 10 min, and then the column was equilibrated with 100% A for 15 min. The MS analysis was performed in data-dependent mode which utilizes HCD to fragment the most abundant 15 ions. Full MS scans were acquired with resolution of 70 K, AGC target of 1e6 and maximum injection time of 250 ms. The MS/MS spectra were generated under normalized collision energy of 30, with isolation width of 2 *m/z*, mass resolution of 17.5 K, AGC target of 1e5, maximum injection time of 150 ms and dynamic exclusion of 45 s.

### Mouse tissue sample preparation

We complied with all relevant ethical regulations for animal testing and research. Animal experiments were conducted following institutional guidelines approved by University of Wisconsin-Madison IACUC. Female C57BL/6J mice were anesthetized, perfused with chilled phosphate buffered saline, decapitated and removed brains. The brains were cut along midline and 100 mg mL^−1^ gelatin in water was used to embed each hemisphere, which was then snap frozen in dry ice. Tissue samples were stored at −80 °C. An Olympus SZX16 stereo microscope (Olympus, Center Valley, PA) was used to take the optical images of tissue sections with bright field illumination.

### TM sprayer for matrix application

A commercial TM sprayer (HTX Technologies, Carrboro, NC) was employed to apply MALDI matrix for tissue MSI. On a cryostat (Thermo Scientific Microm HM 525, Thermo Scientific, Kalamazoo, MI), the gelatin embedded tissues were cryo-sectioned into 12 µm slices at −20 °C, and were then thaw mounted onto ITO glass slides. In a desiccator, the tissue slides were dried for 30 min at room temperature. Thereafter, CHCA matrix were deposited through the robotic TM sprayer system. Key parameters of TM sprayer included, nozzle temperature of 80 °C, moving velocity of 1100 mm/min, eight passes, flow rate of 0.2 mL/min and drying time of 30 s. The sprayed slides were stored in a desiccator after drying at room temperature for 30 min.

### (Sub)AP-MALDI

A SubAP-MALDI (ng) UHR (MassTech, Columbia, MD) was used as a laser ion source and similar laser module can be found in our previous study^47,54^. Notably, this SubAP-MALDI source was featured with a 355 nm high-resolution Nd:YAG laser module along with an integrated ion funnel for improved ion collection and transmission. The typical operation source pressure was 3~5 Torr that made the source SubAP-MALDI. The starting laser parameters (unless otherwise specified) were laser energy of 18.9% and repetition rate of 1000 Hz.

### De-sialylation of glycoprotein transferrin

To study the effects of sialylation on glycoprotein structure, fully sialylated mouse transferrin was de-sialylated by using sialidase of neuraminidase under native conditions. Neuraminidase (Sialidase, from *C. Perfringens* 11585886001 Roche) was supplied by Sigma-Aldrich (St. Louis, MO). First, we dissolved/reconstituted the enzyme to 5 mL water (Sialidase concentration: 1 U/mL). Then, a volume of 100 μL glycoprotein (mouse transferrin, 1 mg mL^−1^ in 100 mM NH_4_OAc, pH 7) was added to a 600 μL EP tube. Then 25 μL sialidase (1 U/mL) was added, while for the control group, 25 μL NaOAc (0.1 M, pH 5) was added instead. After vortex mixing, parafilm sealing, the mixtures were incubated in water bath at 37 °C for 5 h. The de-sialylation was stopped with the addition of 25 μL NaHCO_3_ (0.5 M). After de-sialylation, proteins were buffer-exchanged into 100 mM NH_4_OAc with Amicon Ultra-0.5 Centrifugal Filter Devices (30 K MWCO: UFC503024, Sigma-Aldrich (St. Louis, MO)) following supplier’s standard procedures.

### MALDI-ToF MS for structural probing on large proteins

Large protein samples were tested on a Bruker Rapiflex MALDI-TOF/TOF instrument (Bruker Daltonik, Bremen, Germany). Ion source parameters included: laser energy 89%, frequency 200 Hz, laser 355 nm and each data collection of 10,000 shots. The instrument was calibrated by using bovine serum albumin with the selected peaks of [BSA + 2 H]^2+^ *m/z* 33216, [BSA + H]^+^ *m/z* 66431, [2BSA + H]^+^ *m/z* 132861, and [3BSA + H]^+^ *m/z* 199291. Native proteins were preserved with 100 mM ammonium acetate, while unfolded proteins were obtained through heating in 100 mM ammonium acetate at 95 °C for 10 min by using a dry bath.

### Ion mobility-mass spectrometry

Sialylation effects on glycoprotein transferrin were examined on a commercial ion mobility-mass spectrometer, Waters Synapt G2 (Waters Corp., Manchester, UK). Each glycoprotein sample of approximately 5 μL (~8 μM glycoproteins in 100 mM ammonium acetate) was loaded into a home-made nanospray ion source, and a silver wire of 100 μm thickness was inserted into the borosilicate glass needle for high voltage application. Nanospray voltages ranged between 1.0 and 2.0 kV. The final inner diameter of spray emitters was maintained at ~5 µm, which were pulled from borosilicate glass capillaries using a P-2000 laser-based micropipette puller (Sutter Instruments, Novato, CA, USA). MS instrument was run in positive ion mode and the sampling cone was set at 30 V. The MS cone temperature was 70 °C. The Synapt instrument was tuned to allow preservation and transmission of native proteins and protein interactions. This typically involved elevated pressures in the source region (~6 mbar) and decreasing all focusing voltages (e.g., cone, extractor, and bias voltages). The ion mobility cell was operated at a pressure of ∼3.5 mbar, wave height of 30 V and wave velocity of 400 m/s). The TOF-MS was typically operated over the *m/z* range of 400–8000. Collisional cross-section (CCS) calibration curves were generated using a previously described protocol, and using literature CCS values derived for use with the Synapt instrument platform^9,73^.

### Orbitrap-mass spectrometry

Except the large protein samples that required the extended mass range offered by Bruker RapifleX MALDI-TOF/TOF instrument (Bruker Daltonik, Bremen, Germany; with standard configurations and operations except the modification with NBA), the remaining experiments were carried out on a Thermo QE-HF mass spectrometer (Thermo Fisher Scientific, Bremen, Germany). The SubAP-MALDI source was coupled with QE-HF mass spectrometer for most data acquisition. The Target-ng software (MassTech, Columbia, MD) was used to control the ion source and ion funnel. For spot analysis, an ABI Opti-TOF 192 target plate (Applied Biosystems, Foster City, CA) was employed to allow spotting the peptide standards and matrix solutions. While spiral plate motion was used in spot analysis, constant speed raster motion was used for MSI. The MS was operated under positive polarity mode, with mass range of *m/z* 500–6000 and mass resolution of 60,000 unless otherwise specified. To ensure consistent scan time at each pixel, the maximum injection time was set to 300 ms and automatic gain control target was tuned to 3e6.

### Data analysis

Xcalibur (Thermo Scientific, Bremen, Germany) was used to process QE-HF mass spectra; Waters MassLynx V4.1 (Waters Corp., Manchester, UK) was used for ion mobility-related data processing and Compass flexAnalysis Version 4.0 (Build 14) software (Bruker Daltonik GmbH, Bremen, Germany) was employed to view and analyze large protein data acquired from RapifleX MALDI-TOF/TOF instrument. ImageQuest (Thermo Scientific, Bremen, Germany) and MSiReader (NC State University, Raleigh, NC)^74^ were used for image processing, including image normalization and ion image production. Peaks of protonated ion [M + H]^+^, corresponding sodiated ion [M + Na]^+^, and potassium adduct [M + K]^+^ would be accounted for the same compound. All ions were identified through accurate mass matching from LIPID Metabolites and Pathways Strategy (LIPID MAPS, University of California-San Diego, La Jolla, CA), Sweden peptide database (SwePep, Uppsala University, Uppsala, Sweden) and the Li Lab crustacean neuropeptide databases.

### Calculation functions for NBA labeling efficiency

The labeling efficiencies of NBA tagging were calculated by dividing the number of labeled amines by the number of total amines for each peptide and protein. If there are *n* amines in the peptides, the relative abundances of labeled and unlabeled amines can be expressed as following:

1 $${\mathrm{A}}\left( {{\mathrm{labeled}}} \right) = \mathop {\sum }\limits_{i = 0}^n i \cdot I(\left[ {{\mathrm{peptide}} + {\mathrm{iNBA}}} \right]^ + )$$

2 $${\mathrm{A}}\left( {{\mathrm{unlabeled}}} \right) = \mathop {\sum }\limits_{i = 0}^n (n - i) \cdot I(\left[ {{\mathrm{peptide}} + {\mathrm{iNBA}}} \right]^ + )$$

*I*([peptide + iNBA]^+ ^) stands for the peak intensities of peptide labeled by *i* NBA molecules. Then, the labeling efficiency was defined as following:

3 $${\mathrm{Labeling}}\,{\mathrm{efficiency}}\,\left( \% \right) = \frac{{{\mathrm{A}}({\mathrm{labeled}})}}{{{\mathrm{A}}\left( {{\mathrm{labeled}}} \right) + {\mathrm{A}}({\mathrm{unlabeled}})}}$$

### Reporting summary

Further information on research design is available in the Nature Research Reporting Summary linked to this article.

## Supplementary information

Supplementary Information

Description of Additional Supplementary Files

Supplementary Data 1

Reporting Summary

Source Data

## Data Availability

All data are available from the corresponding author upon reasonable request. The source data for Figs. 3a–d, 4a-b and Supplementary Fig. 3c are provided in a Source Data file.
