# Supplementary material for: Nanosecond photochemically promoted click chemistry for enhanced neuropeptide visualization and rapid protein labeling
Source: Nat Commun. 2019 Oct 16;10:4697. doi: 10.1038/s41467-019-12548-0 (PMC6795811; doi:10.1038/s41467-019-12548-0)
Supplement: Supplementary file 1 — Supplementary Information [file 41467_2019_12548_MOESM1_ESM.pdf]

Supplementary Information

Nanosecond photochemically promoted click chemistry for enhanced neuropeptide visualization and rapid protein labeling

Li et al.

Contents:

Supplementary Figures

Supplementary References

Supplementary Figures

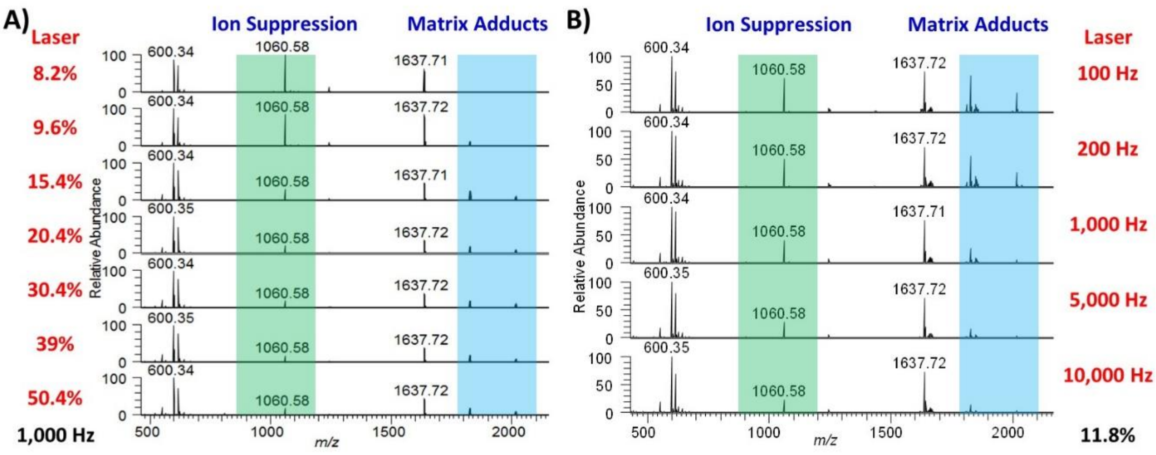

Supplementary Figure 1. **Characterization of SubAP-MALDI**.

A) Optimization of laser energy to evaluate ion suppression effects and matrix adduction effects of SubAP-MALDI using peptide mixture of FMRFamide (*m/z* 600.34), bradykinin (*m/z* 1060.58) and somatostatin (*m/z* 1637.72). **B**) Optimization of firing frequency to evaluate ion suppression effects and matrix adduction effects of SubAP-MALDI using peptide mixture of FMRFamide (*m/z* 600.34), bradykinin (*m/z* 1060.58) and somatostatin (*m/z* 1637.72). Matrix for MALDI, CHCA, 10 mg mL^-1. Laser fluence varied from 8.2% to 50.4% with the laser firing frequency being kept constant at 1,000 Hz. Similarly, laser frequency was varied from 100 Hz to 10,000 Hz with constant laser fluence at 11.8%. Ion suppression effects were characterized by the signal drop of bradykinin (*m/z* 1060.58) and the matrix adduction effects were evaluated using the somatostatin-CHCA bound complexes (1 CHCA-bound and 2 CHCA-bound).

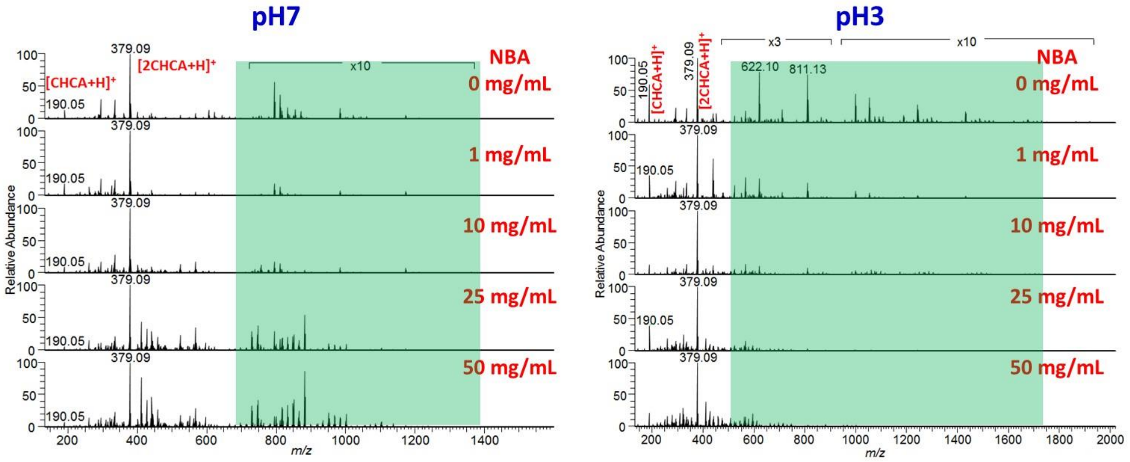

Supplementary Figure 2. **pH-dependent matrix removal by NBA.**

Representative mass spectra for NBA in PBS (1×) buffer solution at lower *m/z* range under two pH conditions (pH 7 and pH 3). Matrix for MALDI, CHCA, 10 mg/mL. As shown above, at pH7, the matrix peaks can be simplified with the gradual elevation of NBA concentration from 0 mg/mL to 10 mg/mL, suggesting the matrix removal effects by NBA-based nsPCR. However, upon further increase in NBA concentration, more complex matrix peaks appeared in corresponding mass spectra. Interestingly, when the pH was switched to acidic (e.g. pH 3), the mass spectra at lower m/z range showed distinct NBA concentration-dependent changing trend. As seen, while the dominate peaks consist of monomer and dimer of CHCA molecule, the other cluster peaks formed from matrix are visibly removed with the gradual increase in concentration of NBA.

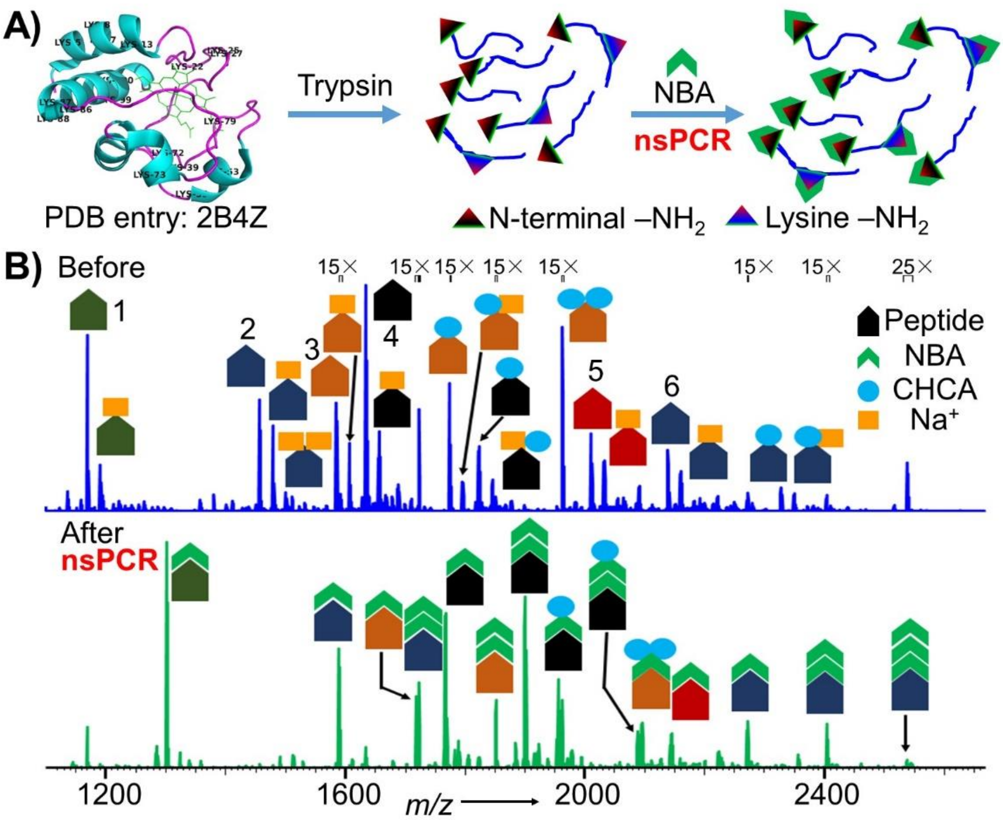

C)

#	Apo-	NS^-	$2NS^-$	$3NS^-$	Sequence	NS^- Label %
1	1168.6185	1301.6342	---	---	TGPNLHGLFGR	88.43
2	1456.6696	1589.6821	1722.6981	---	TGQAPGFSYTDANK	49.45
3	1584.7621	1717.7750	1850.7888	1983.8012	KTGQAPGFSYTDANK	53.06
4	1633.6099	1766.6240	1899.6369	---	CAQCHTVEK (with heme)	76.40
5	2009.9385	2142.9488	2275.9546	---	GITWGEETLMEYLENPK	53.74
6	2138.0299	2271.0390	2404.0493	2537.0573	GITWGEETLMEYLENPKK	55.40

Supplementary Figure 3. Labeling of protein tryptic mixtures via nsPCR.
A) Workflow of NBA-based nsPCR for labeling tryptic cyt c; **B**) Mass spectra for tryptic cyt c fragments (4 pmol) labeling (15.4% laser); **C**) Identified tryptic peptide fragment list with, *m/z*, sequence and labeling efficiency. Each value represents three replicates with RSD less than 5%. Source data are provided in a Source Data file.

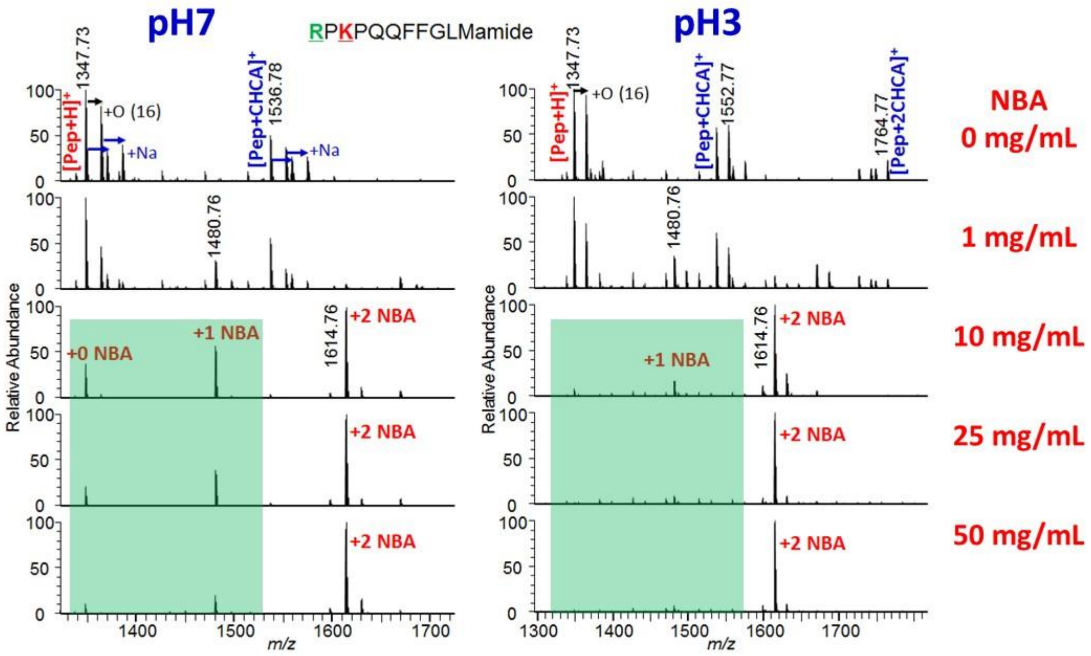

Supplementary Figure 4. **pH effects on NBA labeling efficiency.**

Representative mass spectra for peptide Substance P (RPKPQQFFGLMamide) labeling by using NBA-based nsPCR under two pH conditions (pH 3 and pH 7) with PBS (1×) buffer solution. Matrix for MALDI, CHCA, 10 mg/mL. As observed in the mass spectra, peptide labeling by NBA-based nsPCR progressed in a pH-dependent manner: lower pH condition facilitated the complete stoichiometric tagging of peptides. Under both pH conditions, the peptide spectra derived from PBS buffer contained both sodium adducts and matrix (CHCA) adducts without the use of NBA. In contrast, the spectra have been significantly simplified with the addition of NBA, gradual amine tagging is seen from the appearance of two peaks with the mass shifts of 133 Da and 266 Da, individually. The labeling events show NBA-dependent trend under both pH conditions. The final labeling at pH 7 is not completely stoichiometric where both apo- and one NBA-tagged peaks are present in the spectrum even though at a relative ratio of about 10%. Interestingly, the labeling at pH 3 turns out to be almost complete stoichiometric reaction that the two NBA-tagged peptide peak dominates the spectra (> 99% population) for a peptide (RPKPQQFFGLMamide) with two available tagging sites, N termini and K residue.

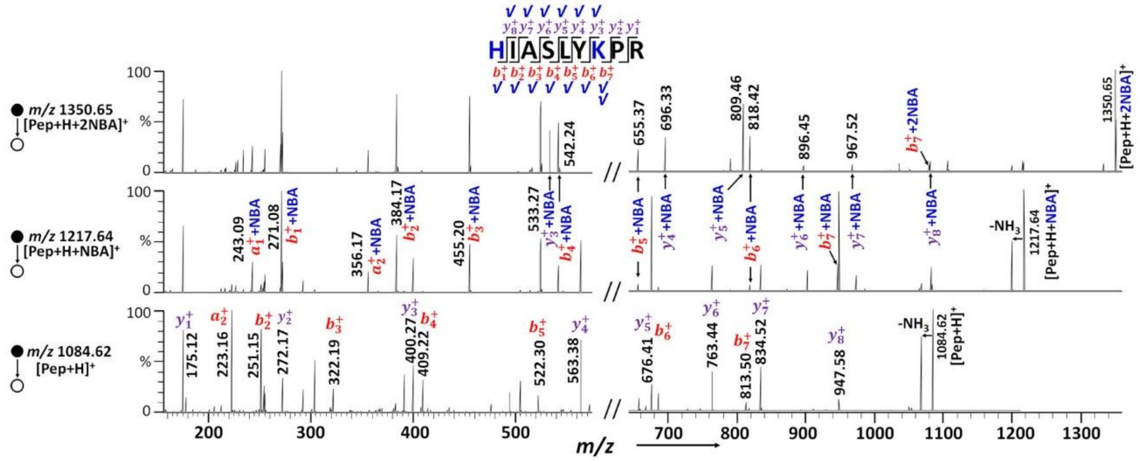

Supplementary Figure 5. **Tandem MS verification of NBA labeling sites.**

Representative tandem MS spectra acquired from HIASLYKPR for apo-form (*m/z* 1084.62) and NBA-tagged form (*m/z* 1217.64 and *m/z* 1350.65). Data were acquired from a SubAP-MALDI-QE HF platform and more details can be found in Online Methods section. Inserted peptide sequencing were also labeled with NBA tagging results by a blue tick mark (1 NBA attached) or two blue tick marks (2 NBA attached).

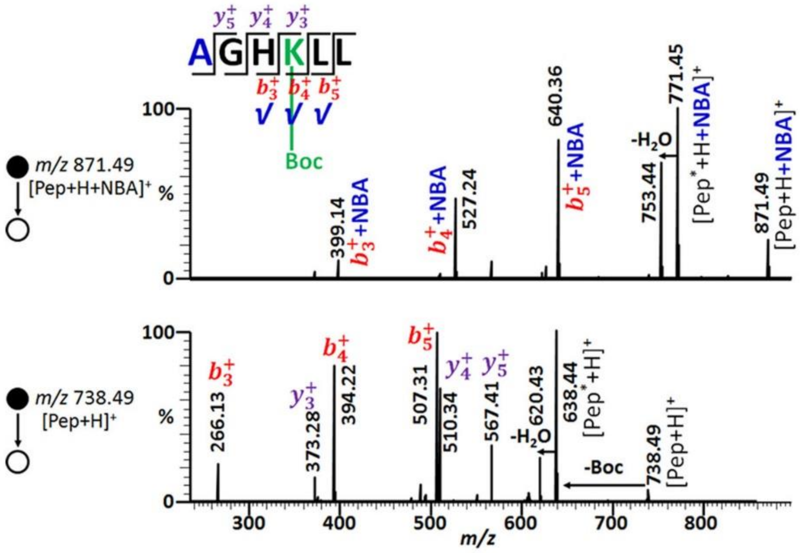

Supplementary Figure 6. **Boc-protection verification of NBA labeling sites.**
Representative tandem MS spectra obtained from AGHK$_{Boc}$LL for apo-form (*m/z* 738.49) and NBA-tagged form (*m/z* 871.49). Data were acquired using a SubAP-MALDI-QE HF platform and more details can be found in Online Methods Section. Inserted peptide sequencing were also labeled with NBA tagging results by a blue tick mark. Boc, tert-butyloxycarbonyl group.

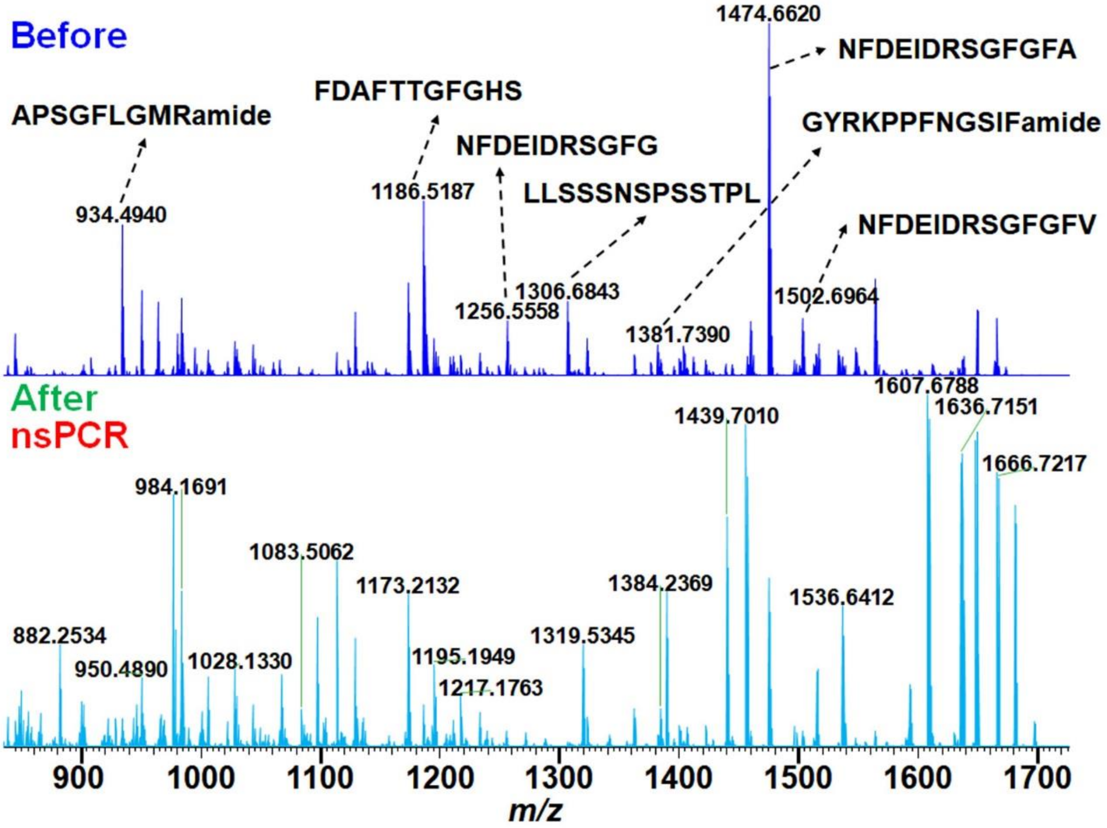

Supplementary Figure 7. **nsPCR labeling of neuropeptide extracts from three crab brains.** In total, 42 neuropeptides have been observed with NS^- labeling after nsPCR (with S/N larger than 10 and mass error window less than 10 ppm during database searching) while 9 of them have been confirmed with LC-ESI-MS/MS with 100% sequence coverage. Only some of the identified neuropeptides are indicated in the figure and a full list of these endogenous neuropeptides can be found in **Supplementary Data 1**.

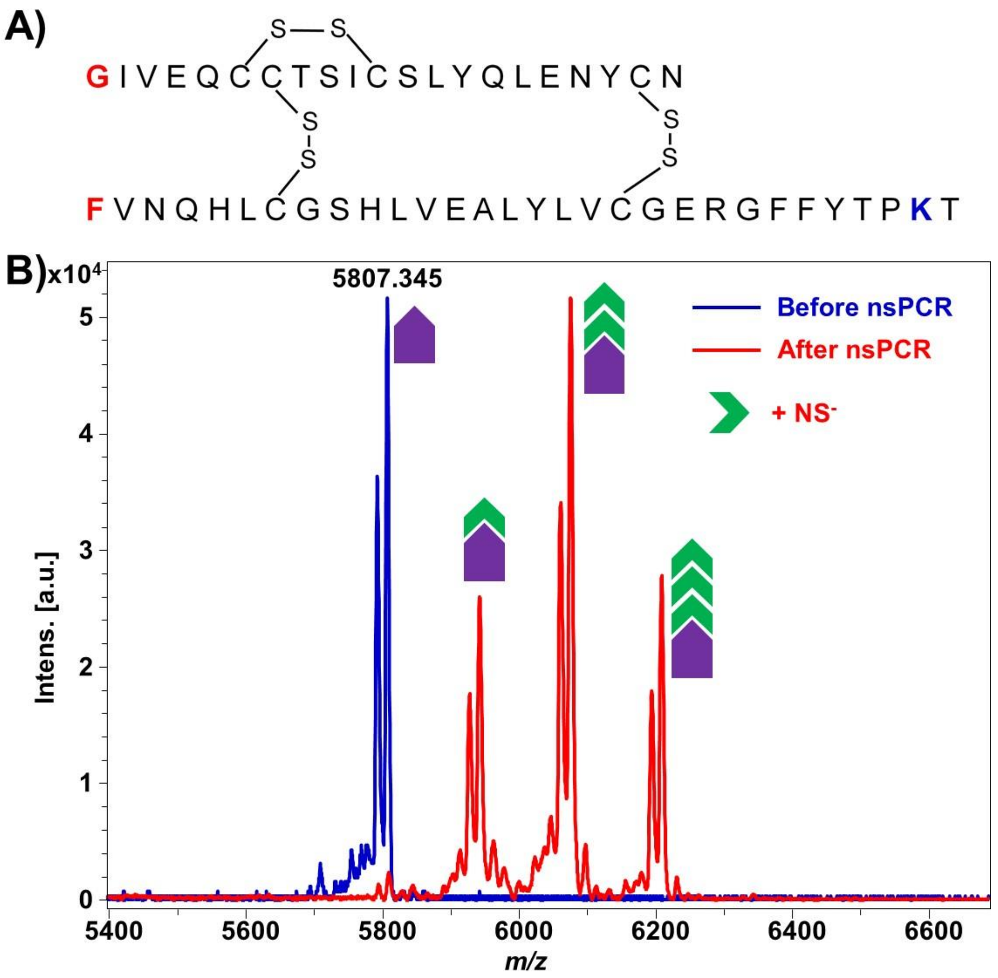

Supplementary Figure 8. **The nsPCR result for intact protein labeling.**

A) Primary sequence of human insulin; **B**) Mass spectra acquired using Bruker RapifleX MALDI-ToF instrument with a laser of 355 nm for intact human insulin photochemical labeling, three labeling residues were identified, corresponding to two N-terminal amine-groups (G and F) and a K side chain-amine group; overall labeling efficiency was higher than 95%. The additional peaks may correspond to the insulin degradation products[1-4] with a mass decrease of 14 Da or an unknown chemical modification.

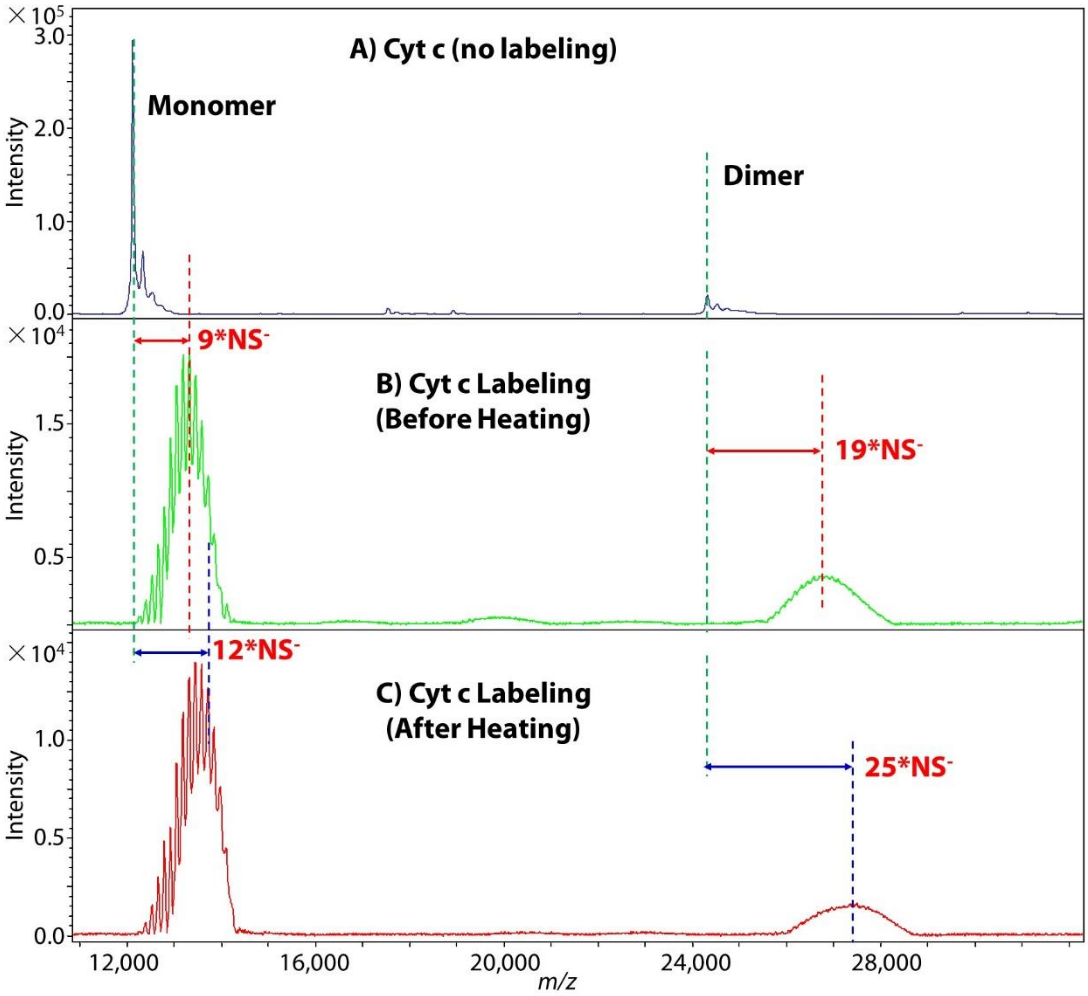

Supplementary Figure 9. **Cytochrome c labeling via nsPCR.**

The nsPCR for labeling and structural probing of cytochrome c using sinapic acid as MALDI matrix. Representative mass spectra for NBA labeling of cytochrome c: **A**, without NBA; **B**, with NBA (before heating); **C**, with NBA (after heating treatment condition) derived from Bruker RapifleX MALDI-TOF/TOF instrument with a laser of 355 nm. The mass increment of protein peak indicates ~9 NS^- and ~12 NS^- for average labeling of native-like and unfolded cytochrome c monomer at the N-terminal amine group and free K-amine groups, respectively; the overall labeling efficiency was approaching 99%. For dimer, the average numbers of labeled NS^- were 19 and 25, respectively. Native proteins were preserved with 100 mM ammonium acetate, while unfolded proteins were obtained through heating in 100 mM ammonium acetate at 95 °C for 10 min.

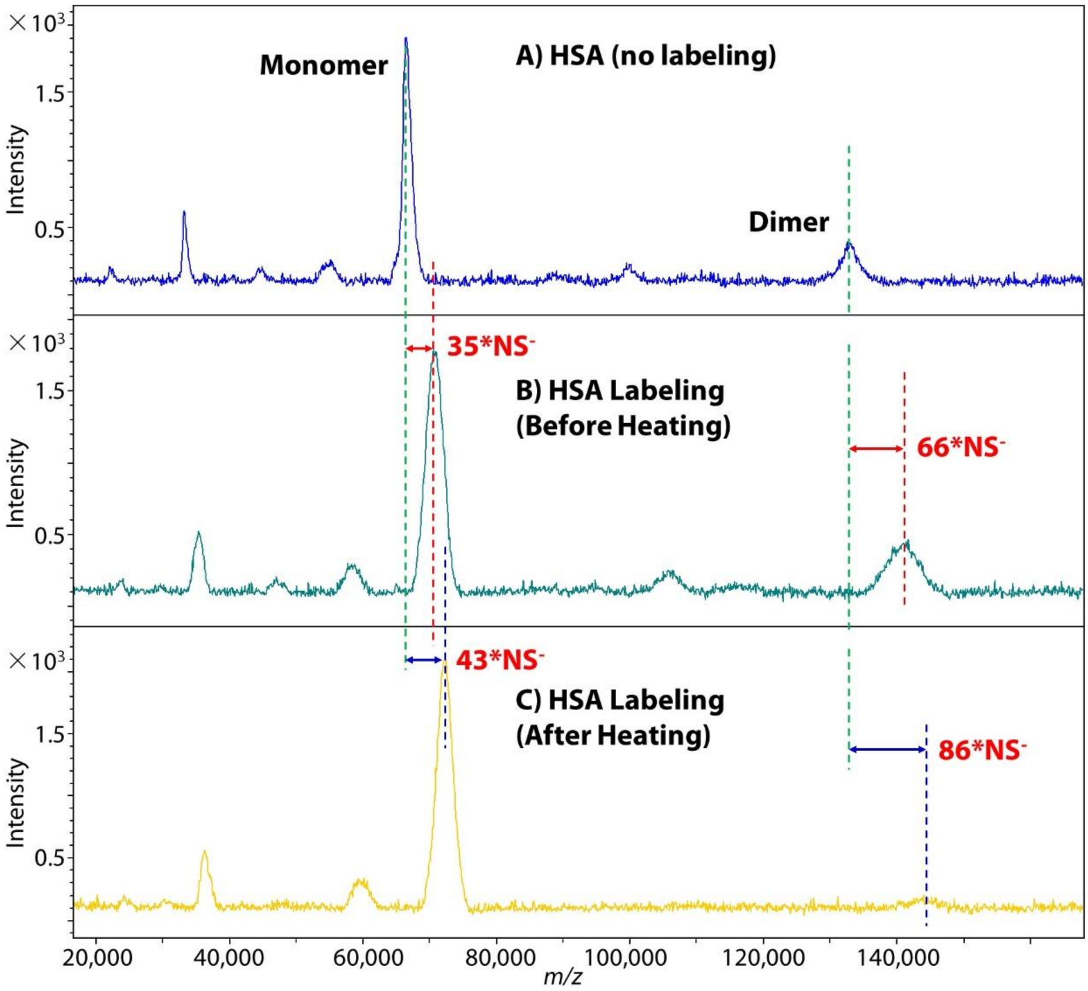

Supplementary Figure 10. **Human serum albumin (HSA) labeling via nsPCR.**
The nsPCR for labeling and structural probing of human serum albumin using sinapic acid as the MALDI matrix. Representative mass spectra for NBA labeling of human serum albumin: **A**, without NBA; **B**, with NBA (before heating); **C**, with NBA (after heating treatment condition) acquired using Bruker Rapiflex MALDI-ToF instrument with a laser of 355 nm. The mass increment of protein peak indicated ~35 NS^- and ~43 NS^- in average labeling of native-like and unfolded human serum albumin monomer at the N-terminal amine group and free K-amine groups, respectively; the overall labeling efficiency was approaching 99%. For dimer, the average numbers of labeled NS^- were 66 and 86, respectively. Native proteins were preserved with 100 mM ammonium acetate, while unfolded proteins were obtained through heating in 100 mM ammonium acetate at 95 °C for 10 min.

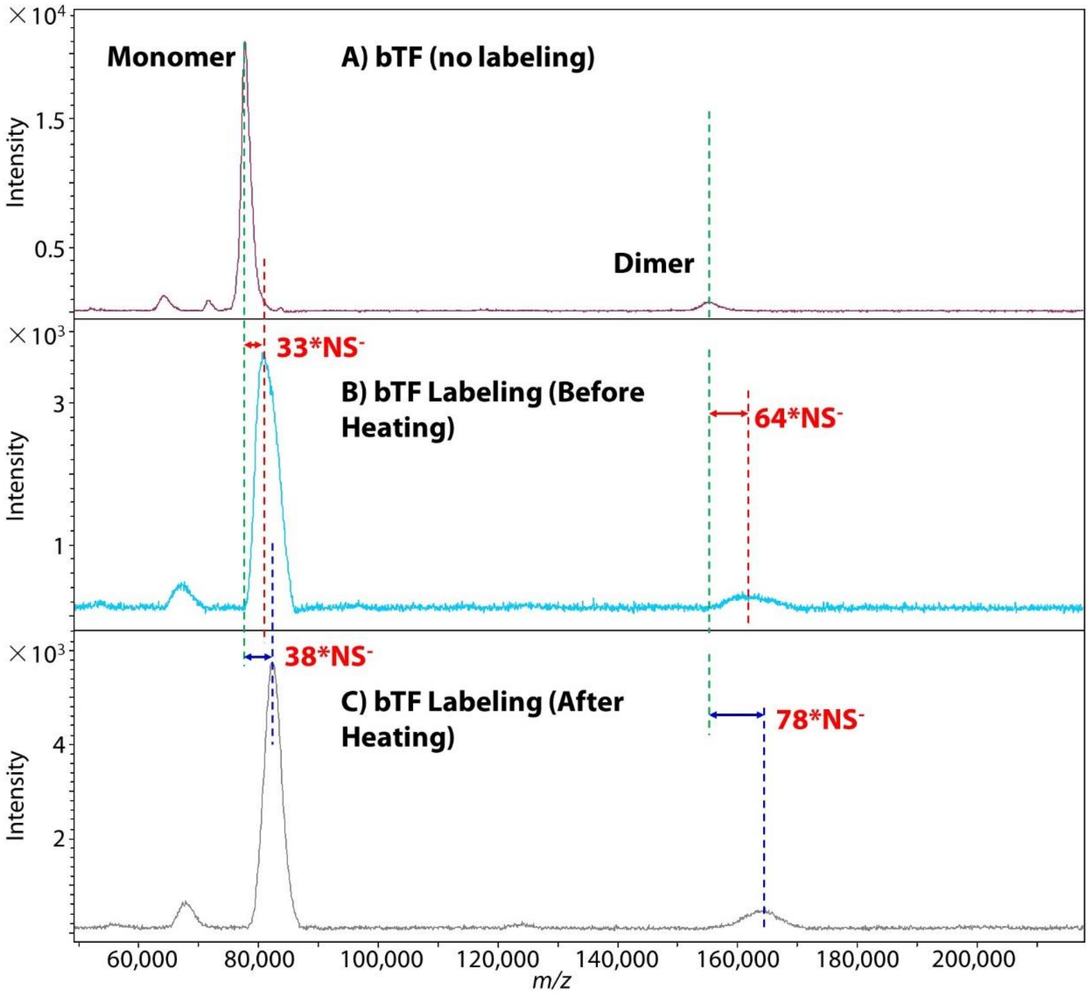

Supplementary Figure 11. **Bovine transferrin (bTF) labeling via nsPCR.**

The nsPCR for labeling and structural probing of bovine transferrin using sinapic acid as the MALDI matrix. Representative mass spectra for NBA labeling of bovine transferrin: **A**, without NBA; **B**, with NBA (before heating); **C**, with NBA (after heating treatment condition) acquired using Bruker Rapiflex MALDI-ToF instrument with a laser of 355 nm. The mass increment of protein peak indicated ~33 NS^- and ~38 NS^- in average labeling of native-like and unfolded human serum albumin monomer at the N-terminal amine group and free K-amine groups, respectively; the overall labeling efficiency was approaching 99%. For dimer, the average numbers of labeled NS^- were 64 and 78, respectively. Native proteins were preserved with 100 mM ammonium acetate, while unfolded proteins were obtained through heating in 100 mM ammonium acetate at 95 °C for 10 min.

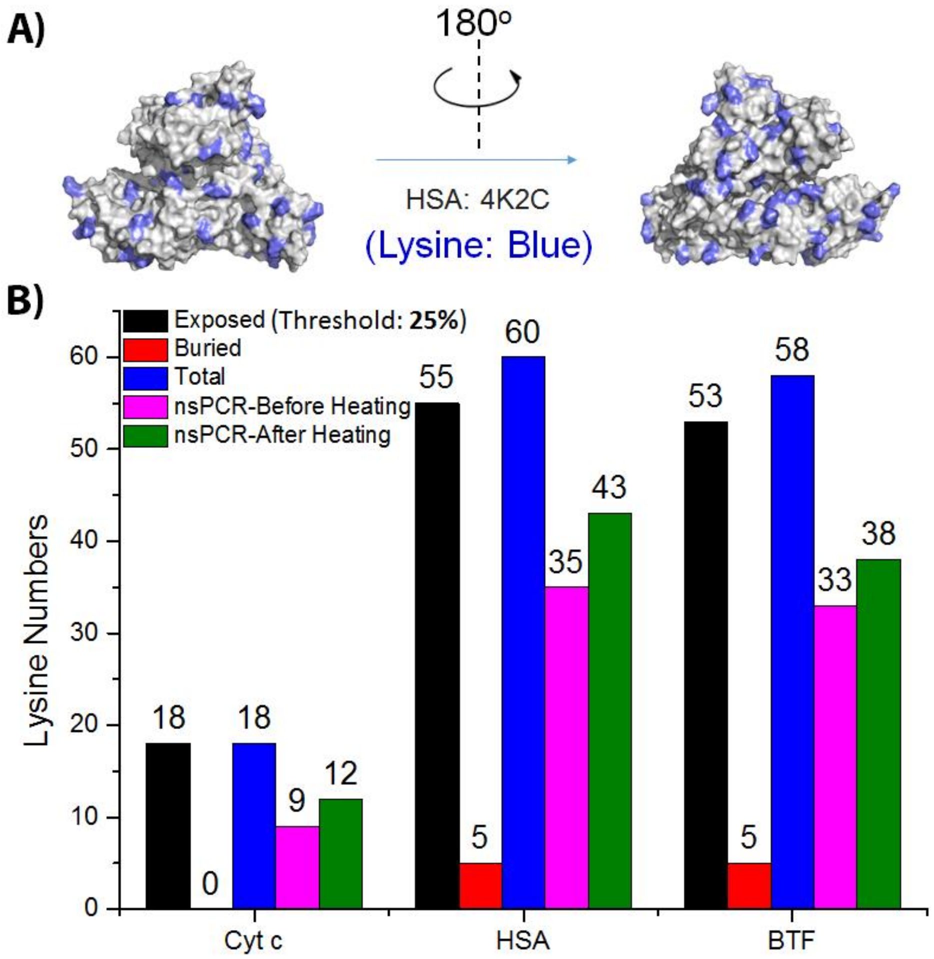

Supplementary Figure 12. **Comparison of surface K number with nsPCR labeling.**
The numbers of surface available amine groups for three proteins (Cyt c, Uniprot ID of P62894; HSA, Uniprot ID of P02768; bTF, Uniprot ID of P02787) and corresponding NBA-based nsPCR labeling results. **A**) Two snapshots from PyMOL of HSA with all surface lysine residues being highlighted with blue. **B**) For the surface accessible lysine residues on proteins, we adopt a tool, NetSurfP-2.0 (REF: Proteins. 2019, 87, 520–527), to calculate the numbers of surface Lys residues. The calculation is based on the default exposure threshold of 25% and the calculated values are then compared with the values that are obtained by using NBA-based nsPCR. The results for three protein monomers in the above figure show that our nsPCR labeling numbers correlate ~50%-60% of the predicted value (with an exposure threshold of 25%) by NetSurfP-2.0. This result suggest that nsPCR may be able to explore lysine residues on protein surface with an exposure more than 40%-50%.

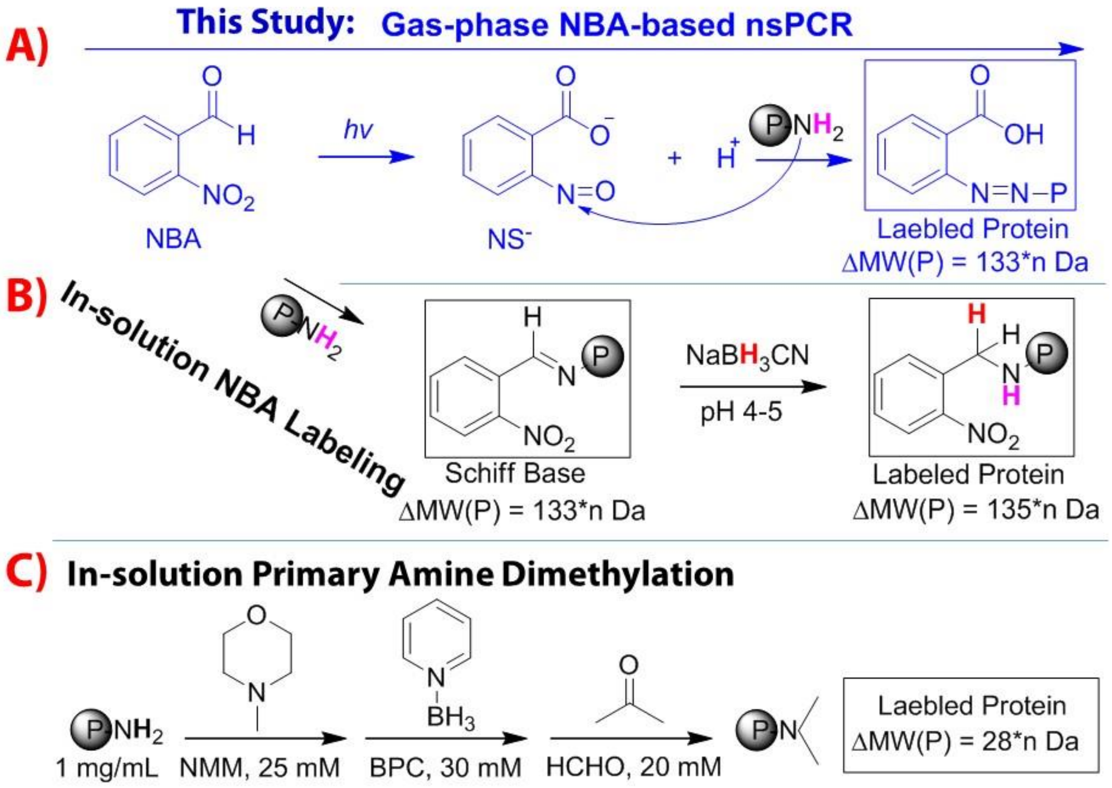

Supplementary Figure 13. **Labeling comparison with three different pathway.**
Schemes for two in-solution labeling reactions as a comparison with the NBA-based nsPCR gas-phase labeling. Three chemical labeling strategies have been proposed including: **A**) Gas-phase, NBA-based nsPCR which has been extensively validated in the main manuscript, with a mass shift of 133 Da for each stoichiometric labeling; **B**) In-solution NBA labeling with the assistance of reducing agent to convert the C-N double bond at the Schiff base (that is unstable under this reaction condition) into stable C-N bond, and the final mass shift was 135 Da for each stoichiometric labeling; **C**) Formaldehyde-assisted dimethylation of primary amine groups of lysine residues, resulting in a mass shift of 28 Da for complete labeling on each amine group. The reactions 2 and 3 were used as control groups and after reaction under the conditions as stated in the schemes, the labeled proteins were then purified using 30 kDa molecular weight cutoff filtration membrane. To avoid any interference originated from MALDI-induced NBA-tagging in the gas phase, the products generated from Reaction 2 were subjected to ESI-MS analysis for validation. Due to relatively complex reaction conditions, all the products from Reaction 3 were subjected to MALDI-MS analysis to avoid any ion suppression effects.

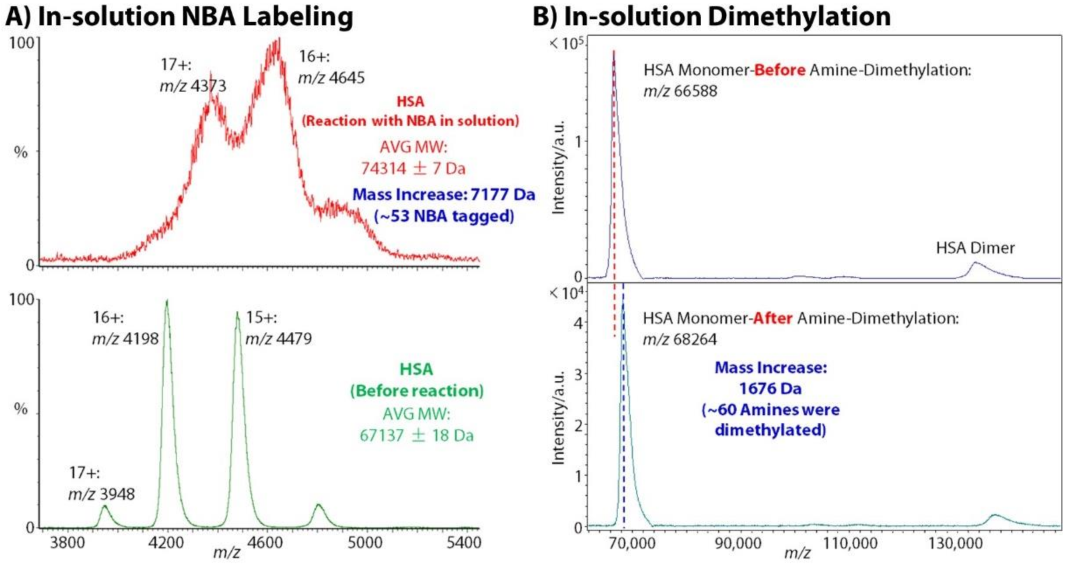

Supplementary Figure 14. **In-solution labeling comparison.**

Representative mass spectra for in-solution labeling by using **A**) NBA and reducing reagent sodium cyanoborohydride and **B**) formaldehyde and reducing reagents (borane pyridine complex) for dimethylation on free amines on Lys residues and N termini. The spectra clearly indicate the mass shift upon in-solution chemical labeling. According to the mass shift value, we find that with the assistance of reducing reagent sodium cyanoborohydride, NBA (panel A) can tag up to 53 primary amine groups in solution, in comparison with 55 exposed (25%) lysine residues and ~35 NBA-tagged primary amine group in the gas phase. In-solution labeling reaction was very close to complete stoichiometry and its numbers appeared to be much higher than gas-phase NBA-based nsPCR. Simultaneously, we found that the dimethylation reaction (panel B) appeared to happen at every primary amine group (60 amines labeled) with this protein (HSA, 61 amines). The direct comparison between Reaction 2 and Reaction 3 suggests the potential steric effect for relatively larger molecule NBA over formaldehyde in terms of approaching free amine groups in proteins especially for those buried inside protein domains. However, we cannot exclude the possibility of protein denaturing or unfolding that might have induced such high labeling numbers during the Reaction 3 due to the organic solvents used and also other harmful reaction conditions.

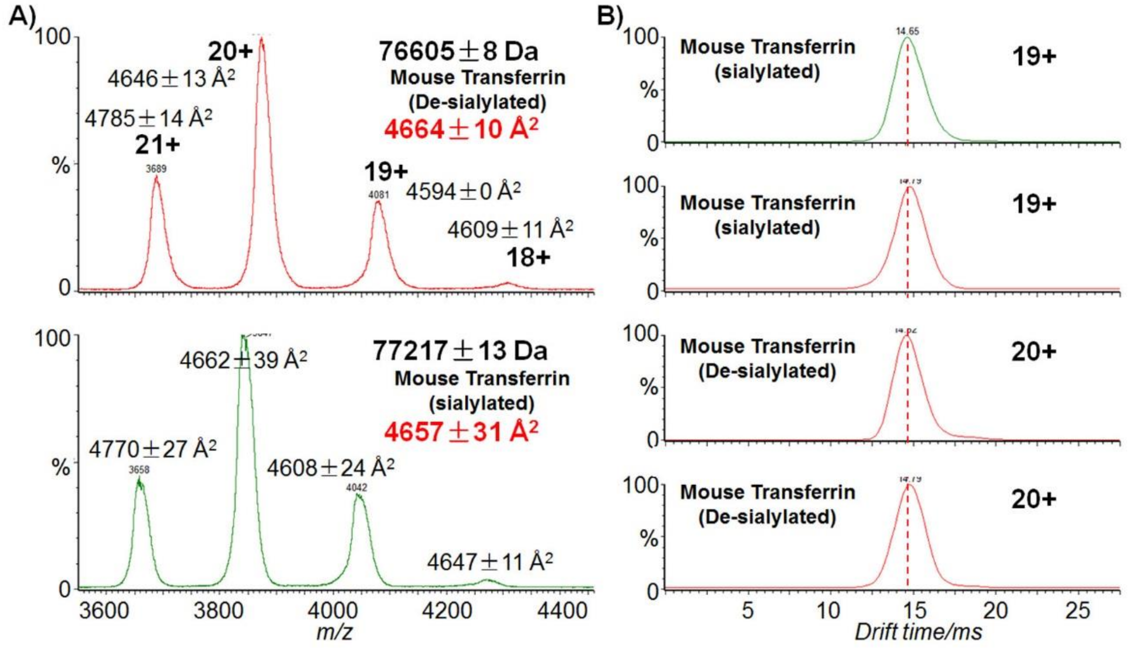

Supplementary Figure 15. **IM-MS measurements of mouse transferrin.**
Ion mobility-mass spectrometry (IM-MS) spectra (**A**) and drift time distributions (**B**) of 19+ and 20+ of sialylated and de-sialylated glycoprotein mouse transferrin. Charge-state dependent collisional cross-section (CCS) values were indicated on corresponding peaks, while the accumulated CCS values according to the charge state populations were shown in red, 4664 ± 10 Å^2 and 4657 ± 31 Å^2 for de-sialylated and sialylated, respectively.

Supplementary References

1. Duckworth, W.C. Insulin degradation: mechanisms, products, and significance. *Endocr. Rev.* **9**, 319-345 (1988).
2. Duckworth, W.C., Bennett, R.G. & Hamel, F.G. Insulin degradation: progress and potential. *Endocr. Rev.* **19**, 608-624 (1998).
3. Hamel, F.G., Peavy, D.E., Ryan, M.P. & Duckworth, W.C. HPLC Analysis of Insulin Degradation Products From Isolated Hepatocytes: Effects of Inhibitors Suggest Intracellular and Extracellular Pathways. *Diabetes* **36**, 702-708 (1987).
4. Seabright, P.J. & Smith, G.D. The characterization of endosomal insulin degradation intermediates and their sequence of production. *Biochem. J.* **320 (Pt 3)**, 947-956 (1996).
