# Supplementary material for: Nanosecond photochemically promoted click chemistry for enhanced neuropeptide visualization and rapid protein labeling
Source: Nat Commun. 2019 Oct 16;10:4697. doi: 10.1038/s41467-019-12548-0 (PMC6795811; doi:10.1038/s41467-019-12548-0)
Supplement: Supplementary file 2 — Description of Additional Supplementary Files [file 41467_2019_12548_MOESM2_ESM.pdf]

Description of Additional Supplementary Files

File name: Supplementary Data 1

Description: The nsPCR-labeled peptide list of extracts from three crab brains. The dataset includes Entry, charge species, sequence information, theoretical m/z, experimental m/z, mass error, signal to noise ratio (S/N), Lysine number, Labeled NBA number and LC-MS/MS confirmation information.
